# Explainability in transformer-based models: methods, tools, and applications in natural language processing and bioinformatics

**DOI:** 10.1093/bib/bbag447

**Published:** 2026-11-12

**Authors:** Naeimeh M Karimi, Mehdi Rezaeian

**Affiliations:** Department of Computer Engineering, Yazd University, Daneshgah Blvd., Safaeieh, Yazd, 8915818411, Iran; Department of Computer Engineering, Yazd University, Daneshgah Blvd., Safaeieh, Yazd, 8915818411, Iran

**Keywords:** transformer-based models, explainability, interpretability, natural language processing (NLP), bioinformatics, biomedical AI

## Abstract

In recent years, Transformer-based models have become state of the art in natural language processing (NLP) and have achieved exceptional performance in a wide range of tasks. Their impact has extended to critical domains such as healthcare and bioinformatics. Transformer models are nonlinear and complex, making it challenging for researchers to understand different aspects of model performance and decision-making processes. Given the widespread use of these models in data processing, particularly in applications related to human health, the need for interpretable and transparent models has become essential. The goal of this paper is to provide a comprehensive review of explainability methods for transformer models, as well as the interpretability tools commonly used in NLP and bioinformatics. First, interpretable methods in different domains are broadly categorized. It then presents a focused discussion on strategies for explaining transformer-based models. By analyzing and comparing these methods, their strengths, applicability to transformers, and limitations are highlighted. As model complexity increases, understanding model behavior becomes more challenging. Explainability techniques can help address this gap and support tasks such as error diagnostics, model improvement, and other applications. Greater clarity in a model’s decision-making process can increase user confidence. Interpretable models are particularly important in critical applications such as drug design, where they can support deeper investigation and facilitate new discoveries. Finally, this paper outlines potential directions for future research.

## Graphical Abstract

**Graphical Abstract:**
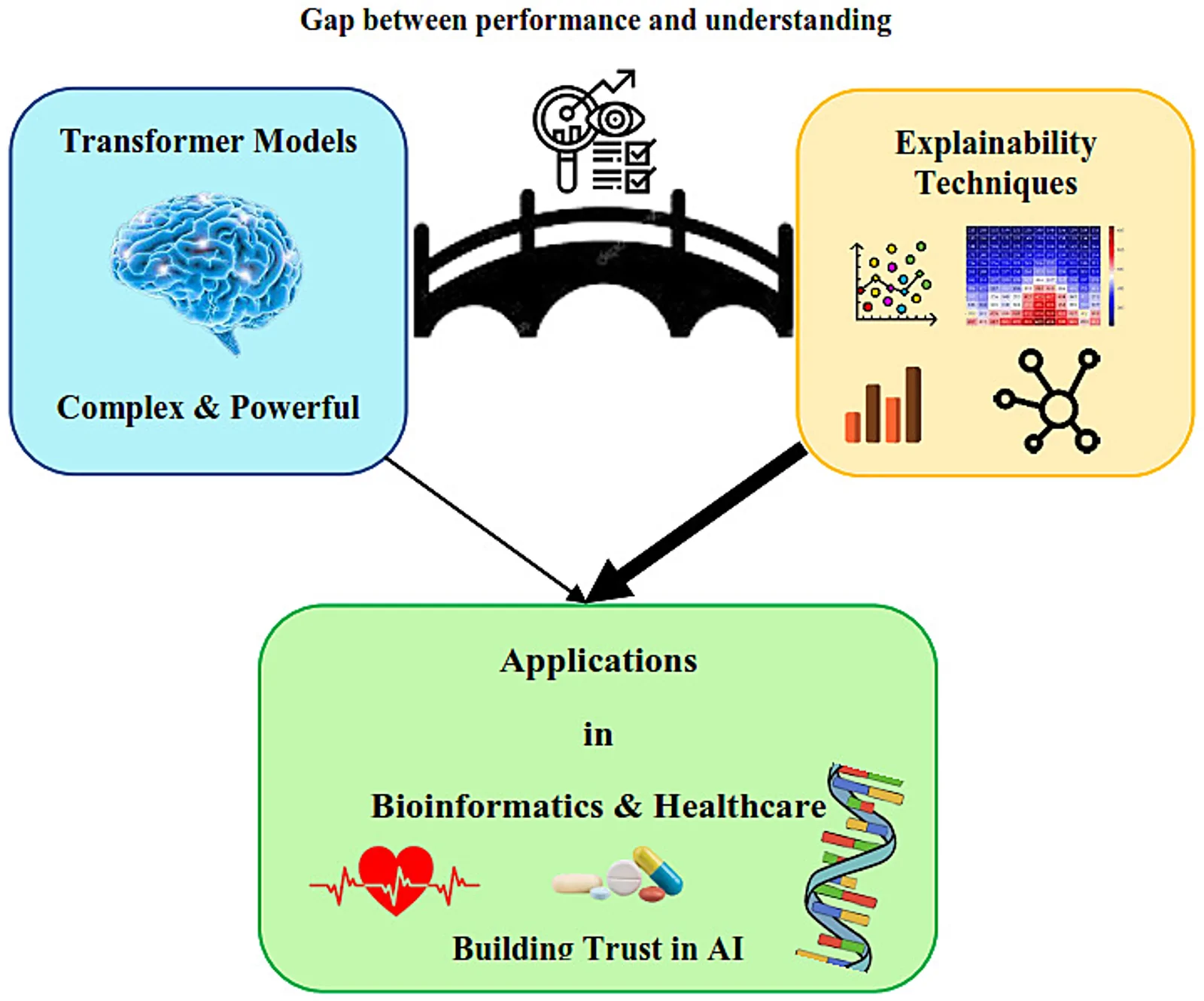

## Explainability or interpretability?

In the literature, the terms interpretability and explainability are often used in varying contexts. While some treat them as synonyms, a subtle distinction exists. Interpretability methods generally focus on understanding direct cause-and-effect relationships and providing a transparent overview of a model’s logic. For example, attention weights can be visualized to identify emphasized parts of a sentence. However, relying solely on these weights may not always provide a faithful representation of the model’s internal reasoning. In many cases, understanding how and why these weights influence final predictions requires dedicated explainability methods to clarify their underlying behavior and faithfulness.

Essentially, while interpretability relates to the inherent transparency of a model’s operations, explainability seeks to uncover the deeper, often abstract, reasoning behind its decisions. In practice, these terms are frequently used interchangeably, and this review examines both perspectives with a primary focus on explainability. As illustrated in Fig. 1, the choice between these approaches is closely linked to model complexity. Simpler models inherently allow for better interpretability and a direct understanding of input–output mappings. However, as model complexity increases—as seen in large-scale Transformers—specialized explainability methods become essential to analyze and verify the model’s internal mechanisms.

**Figure 1:**
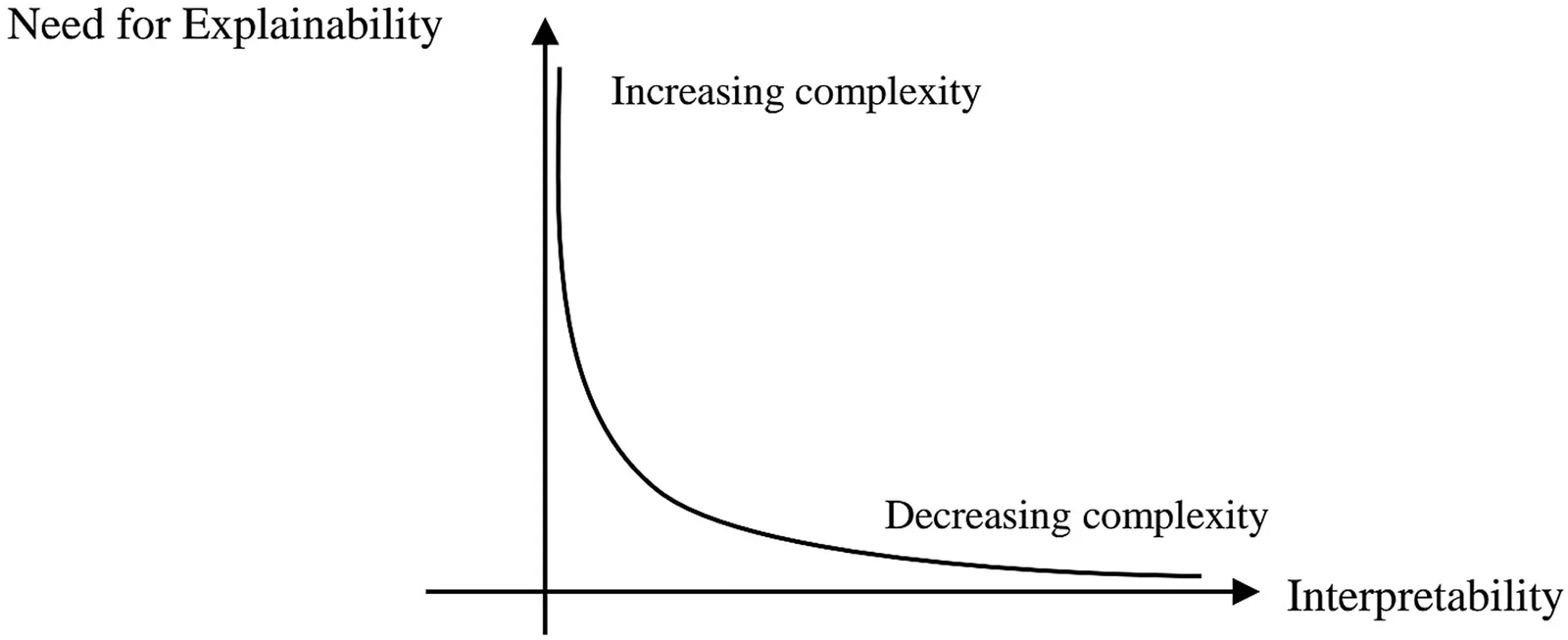
Conceptual relationship between model complexity, interpretability, and explainability. As model complexity increases, intrinsic interpretability decreases, and the need for explainability methods increases.

## Why is interpretability important?

Interpretable models enhance user trust and acceptance among domain experts due to their transparency. Research indicates that user understanding of machine learning systems increases significantly with interpretability [1–3]. In healthcare, where diagnostic or predictive models directly influence patient outcomes, interpretability is crucial for ensuring that clinical decisions are grounded in sound reasoning [4–6]. Furthermore, linguistic models can inherit unwanted biases from training data. Interpretability aids in identifying these biased patterns, enabling corrective actions to mitigate discrimination [1, 2]. Bias often arises from unsuitable artificial intelligence (AI) methods, incorrect parameter settings, or imbalanced data—factors that may remain hidden without transparent mechanisms. By facilitating the identification of these features, interpretability helps reconcile discrepancies between model outputs and expert-approved clinical targets [7, 8]. Explainability provides deeper insights into the features driving model predictions, which is especially vital in bioinformatics. Given the sensitive nature of biological data and the high stakes of clinical applications, explainability allows researchers to verify that predictions are based on relevant biological signals rather than noise, thereby ensuring the reliability of downstream decisions [7, 9, 10]. This transparency enables users to make informed choices rather than blindly following AI-generated content. Moreover, explainability is a cornerstone of responsible AI; rather than making the AI system “responsible” for decisions, it ensures human accountability by providing the necessary transparency to align AI behavior with ethical standards and social values [2, 3, 11]. Recent discussions also emphasize the faithfulness of these explanations, ensuring they accurately reflect the model’s internal logic rather than providing merely plausible but misleading justifications [2, 3]. Finally, explainability supports scientific discovery (TGDS (Theory-guided data science)) by revealing novel patterns in complex datasets across fields such as biomarker discovery and quantum chemistry [12]. Figure 2 visually summarizes these key motivations.

**Figure 2:**
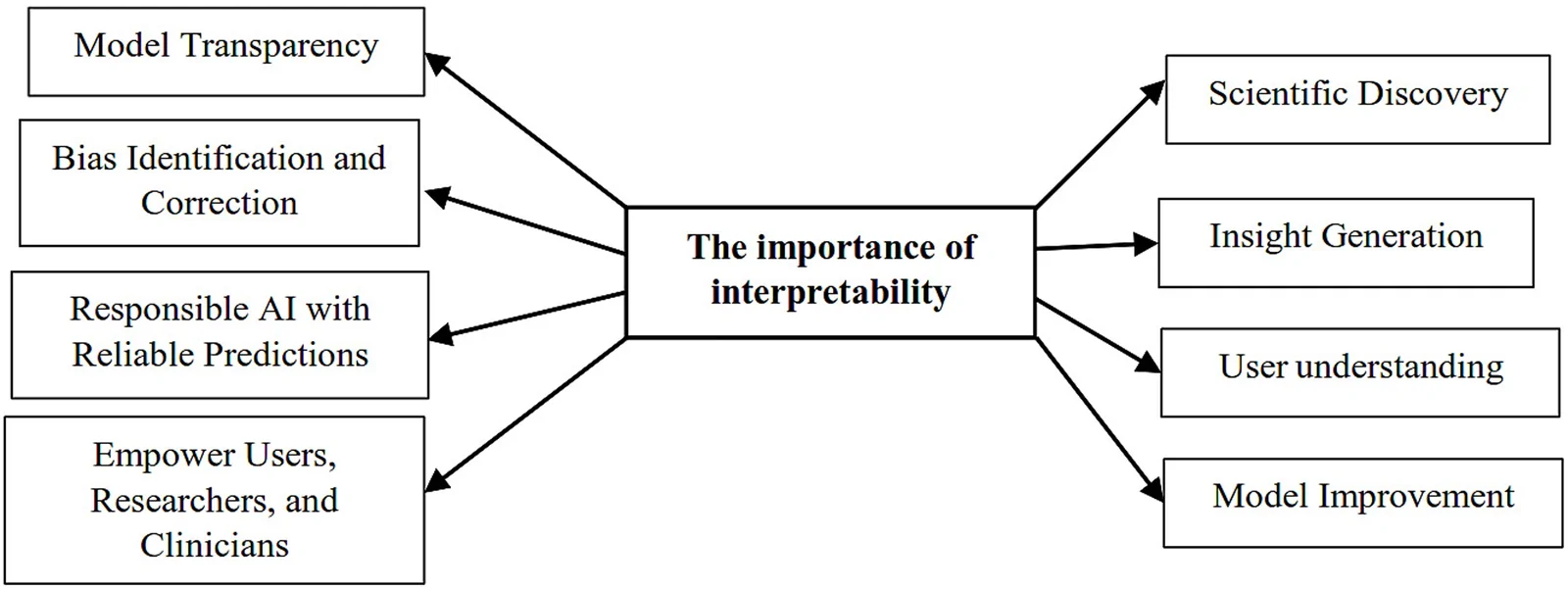
Key motivations for interpretability in AI systems, including transparency, bias detection, responsible AI, user understanding, model improvement, and scientific discovery.

## Introduction

Biomedical data science is an interdisciplinary field that uses principles and techniques from data science, statistics, computer science, and domain-specific biomedical knowledge to analyze and extract insights from biomedical data (Fig. 3). AI techniques such as machine learning [13], deep learning [9], natural language processing (NLP) [14], and computer vision [6, 15] are widely used in biomedical data science. AI and data science are complementary in analyzing data, extracting hidden patterns, and solving complex problems across various domains. AI has played an essential role in the evolution of biomedical research and healthcare by providing innovative solutions in predictive modeling, diagnosis and medical imaging, drug discovery [16, 17] and development [18, 19], EHR (Electronic health records) analytics [8], and bioinformatics. Researchers in this field systematically collect diverse data, including genomics, proteomics, medical imaging, electronic health records, clinical trial data, and other related datasets [20]. However, the rapid and large-scale analysis of these data remains a fundamental challenge that AI aims to address.

**Figure 3:**
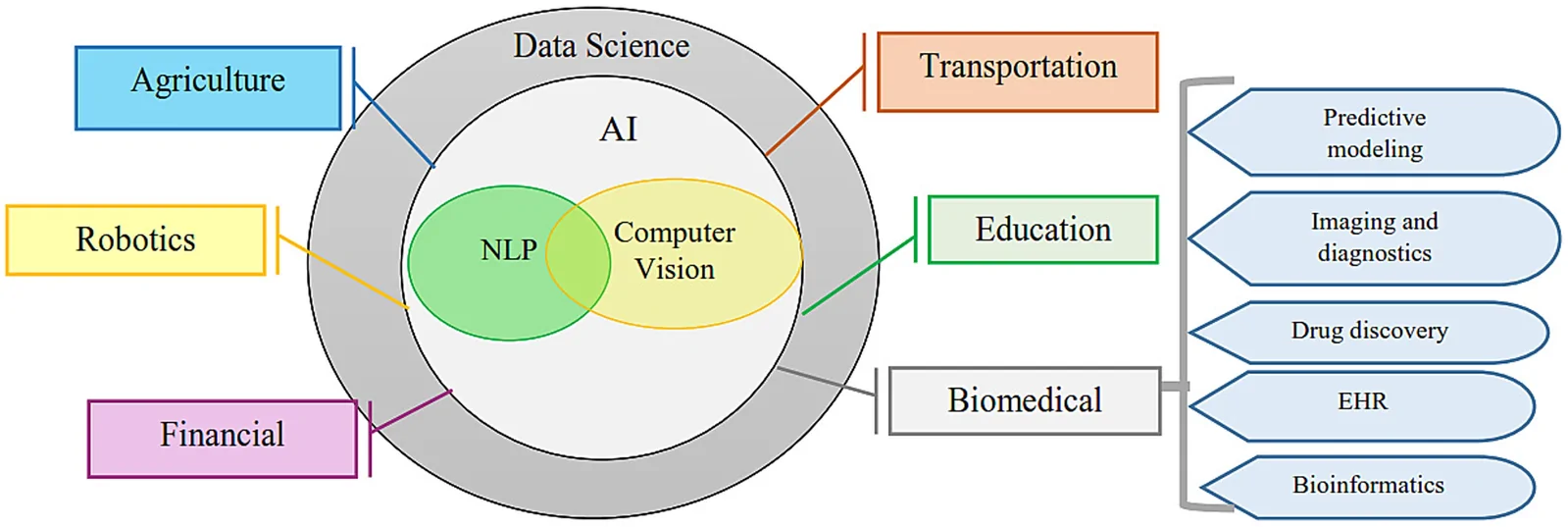
An overview of the relationship between artificial intelligence (AI) and data science.

To address this challenge, graph-based deep learning has shown remarkable potential in modeling complex biological dependencies. Recent state-of-the-art frameworks have significantly advanced molecular interaction predictions, such as circRNA–miRNA interactions (e.g. DeepCMI [21]) and DTI (Drug–target interaction) discovery (e.g. PPAEDTI [16]). Furthermore, in the field of single-cell transcriptomics, methods like scMCGraph [22] and scGraphDap [23] have utilized graph neural networks to enhance cell type annotation and infer cell–cell communications within tumor microenvironments. To bridge the gap between performance and biological understanding, more recent approaches such as scProGraph [24] have integrated interpretability into graph models, enabling the discovery of functional gene subgraphs and regulatory mechanisms.

NLP is a subfield of AI that focuses on enabling computers to analyze, understand, and generate human language [25]. In recent years, NLP technologies have significantly improved text processing and enabled a wide range of applications in bioinformatics, including biomedical text mining [26], sequence analysis [27], protein and nucleotide annotation [28], and ontology construction [29]. These technologies have evolved through the development of white-box, black-box, and hybrid methods. The primary trade-off involves balancing predictive performance and interpretability. Examples include rule-based models, decision trees, hidden Markov models, and logistic regression, which are often referred to as white-box techniques due to their transparent structure. As data representations became more complex, embedding vectors were introduced as feature representations, leading to more sophisticated models such as deep learning architectures and, subsequently, transformer models. Hybrid methods attempt to combine the strengths of both approaches by integrating interpretable components with more complex black-box architectures. A summary of these techniques is provided in Table 1.

**Table 1:** Classification of NLP techniques based on model interpretability.

	Techniques	Description (for example)
**White box techniques**	Rule-based techniques	These methods operate based on explicit if–then rules, making the decision process transparent and easy to interpret. They have been used in tasks such as sentiment analysis [30, 31] and knowledge-based biological classification [32].
	Decision Trees	Decision trees are interpretable models composed of decision nodes and leaf outputs. They have been applied to sentiment analysis in NLP [30] and to various bioinformatics tasks [33].
	Hidden Markov Models (HMMs)	HMMs are probabilistic models for sequential data and are particularly suitable for sequence-labeling tasks. They have been applied to part-of-speech tagging in NLP [34] and to applications such as sequence alignment, gene annotation, and similarity search in bioinformatics [35, 36].
	Logistic Regression	Logistic regression is a simple and interpretable classification model commonly used in sentiment classification [37, 38] and for tasks such as disease prediction and gene-expression analysis in bioinformatics [39].
**Black box techniques**	Deep learning models	This category includes architectures such as RNNs, LSTMs, and transformer-based models, which can learn complex patterns from large datasets but are difficult to interpret. They are widely used in NLP [40] and bioinformatics [9, 10].
	Representation learning/embeddings	Word embeddings (such as Word2Vec and GloVe) or text embeddings (e.g. BERT embeddings) demonstrate semantic relationships between words. Embeddings contain latent features extracted from the text, which can serve as features for downstream NLP tasks [40] or bioinformatics [41, 42].
	Neural language models	Neural language models (e.g. GPT models) learn probability distributions over word sequences and can generate contextually relevant text. They can be fine-tuned for various NLP tasks [18, 40, 43].
	Ensemble models	Ensemble techniques combine the outputs of multiple models to improve predictive performance [44]. Methods such as XGBoost and LightGBM [45] effectively capture nonlinear relationships in data and have shown strong performance in NLP and bioinformatics applications [44, 46].
**Hybrid techniques**	Rule-based systems with embeddings	In these models, embeddings extracted from deep learning models are used as features within rule-based systems. This allows combining the expressive power of representation learning with the interpretability of symbolic rules [47, 48].
	Attention Mechanisms	Attention functions as an interpretable component inside transformer architectures and is widely used in NLP and bioinformatics [49]. By assigning different weights to input elements, attention provides insights into which parts of the data contribute more to the model’s predictions.

White-box, black-box, and hybrid techniques can be broadly distinguished based on their interpretability. White-box models (e.g. rule-based systems and decision trees) provide transparent decision-making processes that can be directly inspected. Black-box models (e.g. deep learning and neural language models) often achieve higher predictive performance but lack interpretability due to their complex internal representations. Hybrid techniques combine interpretable components with black-box architectures to balance transparency and predictive performance. These general definitions are consistent with the interpretability literature [50, 51].

This study focuses on the interpretability of transformer models and attention mechanisms. Transformer-based architectures have become highly influential due to their strong performance in NLP, computer vision, and several bioinformatics applications. These models are particularly effective at capturing complex patterns in large-scale data. However, their extensive parameterization and multi-layer self-attention computations make them difficult to interpret. Therefore, understanding and explaining the decision-making process of transformer models is an important research challenge [52]. In Fig. 4 and the equations below, we conceptually compare different model categories in terms of interpretability, predictive accuracy, and training time.


$$\left.\begin{array}{c}\mathrm{if}\ {\mathrm{I}}_{\mathrm{BB}}\mathrm{be}\ \mathrm{Interpretability}\ \mathrm{of}\ \mathrm{the}\ \mathrm{black}\ \mathrm{box}\\ {}\mathrm{if}\ {\mathrm{I}}_{\mathrm{WB}}\mathrm{be}\ \mathrm{Interpretability}\ \mathrm{of}\ \mathrm{the}\ \mathrm{white}\ \mathrm{box}\\ {}\mathrm{if}\ {\mathrm{I}}_{\mathrm{HB}}\mathrm{be}\ \mathrm{Interpretability}\ \mathrm{of}\ \mathrm{the}\ \mathrm{hybrid}\ \mathrm{box}\end{array}\kern0.75em \right\rangle\ {I}_{WB}>{I}_{HB}>{I}_{BB} $$

$$\left.\begin{array}{c}\mathrm{if}\ {\mathrm{A}}_{\mathrm{BB}}\mathrm{be}\ \mathrm{Accuracy}\ \mathrm{of}\ \mathrm{the}\ \mathrm{black}\ \mathrm{box}\\ {}\mathrm{if}\ {\mathrm{A}}_{\mathrm{WB}}\mathrm{be}\ \mathrm{Accuracy}\ \mathrm{of}\ \mathrm{the}\ \mathrm{white}\ \mathrm{box}\kern1em \\ {}\mathrm{if}\ {A}_{\mathrm{HB}}\mathrm{be}\ \mathrm{Accuracy}\ \mathrm{of}\ \mathrm{the}\ \mathrm{hybrid}\ \mathrm{box}\end{array}\right\rangle\ {A}_{HB}\ge {A}_{BB}>{A}_{WB}$$

(1) \begin{eqnarray*} \left.\begin{array}{c}\mathrm{if}\ {T}_{\mathrm{BB}}\mathrm{be}\ \mathrm{train}\ \mathrm{time}\ \mathrm{of}\ \mathrm{the}\ \mathrm{black}\ \mathrm{box}\\ {}\mathrm{if}\ {\mathrm{T}}_{\mathrm{WB}}\mathrm{be}\ \mathrm{train}\ \mathrm{time}\ \mathrm{of}\ \mathrm{the}\ \mathrm{white}\ \mathrm{box}\\ {}\mathrm{if}\ {\mathrm{T}}_{\mathrm{HB}}\mathrm{be}\ \mathrm{train}\ \mathrm{time}\ \mathrm{of}\ \mathrm{the}\ \mathrm{hybrid}\ \mathrm{box}\end{array}\kern0.75em \right\rangle\ {T}_{HB}\ge {T}_{BB}>{T}_{WB} \end{eqnarray*}

**Figure 4:**
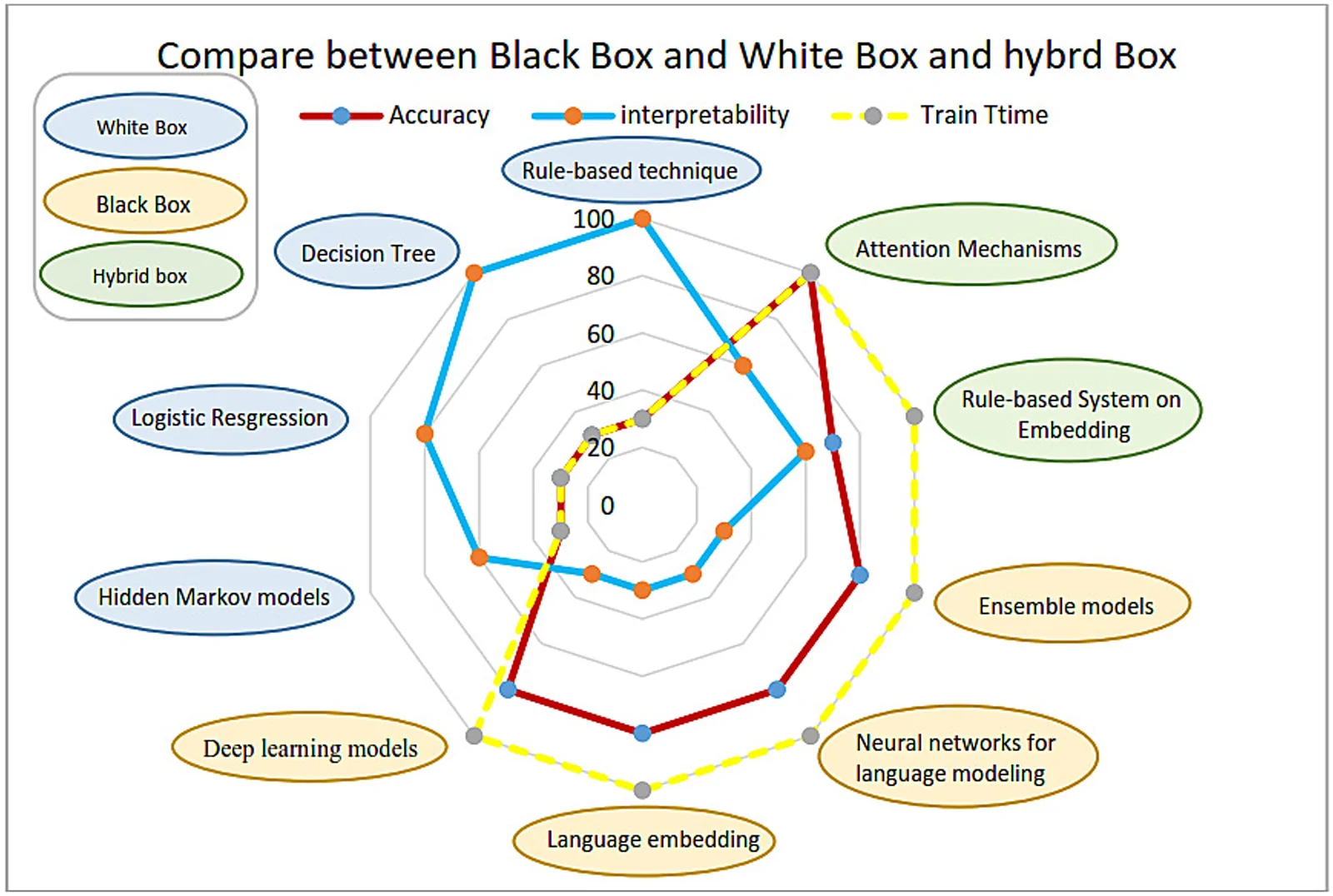
A symbolic (nonquantitative) representation of the relationship between black-box, white-box, and hybrid models in terms of accuracy, interpretability, and training time. The radial values are illustrative and do not correspond to measured units.

## Background

### Interpretability and explainability: main perspectives

Machine learning and deep learning algorithms have widely permeated everyday human life, significantly impacting various aspects of our daily interactions with technology. These algorithms are applied in numerous fields, including medicine, crime risk assessment, autonomous vehicle management [3], economics, and other domains. Several keynotes on these algorithms, including transparency, interpretability, reliability, and fair prediction, are paramount. The need for interpretable AI systems is growing, and researchers are investigating interpretability from various perspectives. We will explore several different viewpoints in the following.

#### Intrinsic interpretable model versus *post hoc* explainable model

Intrinsic methods (e.g. linear models, decision trees, GAMs (Generalized additive models)) are inherently interpretable, namely, interpretation embedded in the model’s design. In contrast, *post hoc* methods (e.g. gradient-based saliency, feature visualization) explain a model after training [3].

#### Local versus global

Local methods explain the model’s behavior for specific instances [3] and highlight the most influential features (e.g. saliency maps [53], LIME [54], SHAP [55, 56]). Global methods summarize whole patterns in the entire dataset and help identify general feature importance (e.g. via visualization techniques or SHAP). Notably, SHAP can be applied in both local and global contexts depending on the analysis.

#### Model-agnostic versus model-specific

Two other views on interpretability are model-agnostic and model-specific methods [3]. Model-agnostic methods focus on understanding the relationship between inputs and outputs without considering the specific details of the model. Examples of methods presented as model-agnostic are LIME and SHAP. In contrast, model-specific methods consider each model’s details, structure, and parameters and provide deeper insight into its inner workings.

#### Norm-based analysis versus weighted-based analysis

Most methods developed for explaining AI algorithms rely on the weights obtained. Any input vector with a higher weight receives more attention. The norm of the input vector contains information and can aid in better explaining the model [57]. In other words, the attention to a specific part of the input is determined by two quantities: the attention weight and the magnitude of the input vector. This section will be examined in detail in the following parts.

#### Classification of different viewpoints into two categories of interpretability and explainability

Researchers have proposed various categorizations for interpretability [3, 58–60]. Figure 5 presents a general classification of different aspects of model interpretability. In general, simple and inherently transparent models are associated with interpretability, whereas more complex models often require *post hoc* explainability methods to provide insights [3]. At the next level, two approaches, intrinsic interpretability and *post hoc* explainability, are considered [3]. Intrinsically interpretable models are generally associated with the interpretability category, while *post hoc* explainable models are associated with the explainability category [3]. Both categories—interpretability and explainability—can provide insights in both local and global forms [3]. However, interpretability is more commonly linked to global understanding, whereas explainability is more frequently used for local analysis. Regarding model-agnostic and model-specific approaches, both interpretability and explainability include examples of each, although explainability methods often rely more heavily on model-agnostic techniques. The last two methods, weight-based and norm-based, are more commonly associated with the explainability category [57].

**Figure 5:**
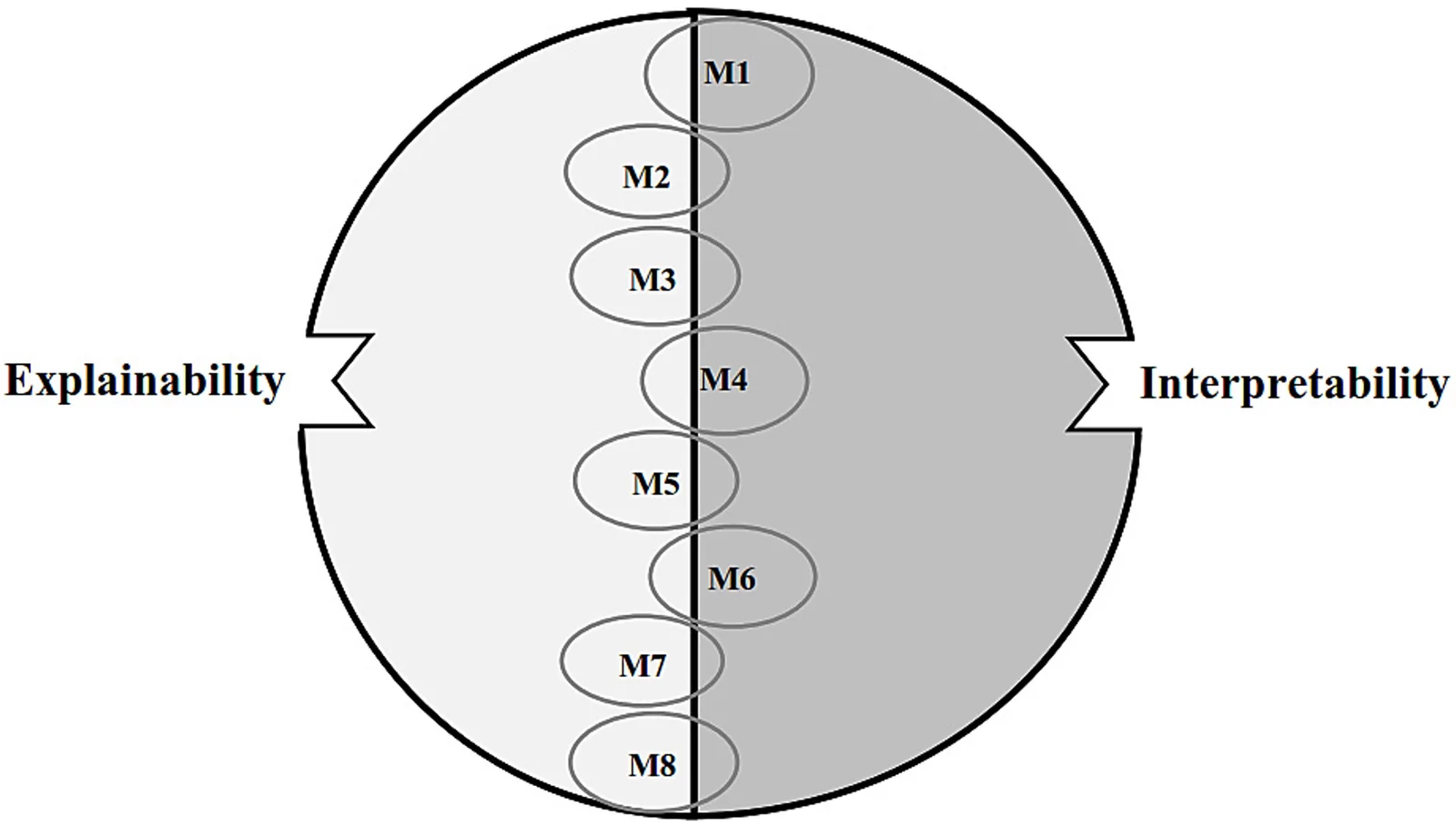
A brief overview of various interpretability categories for artificial intelligence algorithms. M1: Intrinsic Interpretable Models, M2: Post-Hoc Explainable Models, M3: Local, M4: Global, M5: model-agnostic, M6: model-specific, M7: Weighted-Based, M8: Norm-Based.

### Gradient-based methods

Gradient-based methods are among the main approaches to interpretability in deep learning models. In these methods, by computing the derivative of the model output with respect to the input features, the sensitivity of the model to small changes in the input is measured [53]. In linear models, this relationship is relatively simple; however, in nonlinear models, a first-order Taylor expansion is usually used to approximate the local behavior of the model. This approximation is only valid in small neighborhoods around the input, and applying large changes to the input may lead to misleading interpretations. Gradient-based methods can be divided into two categories: sensitivity-based methods and attribution-based methods (Fig. 6).

**Figure 6:**
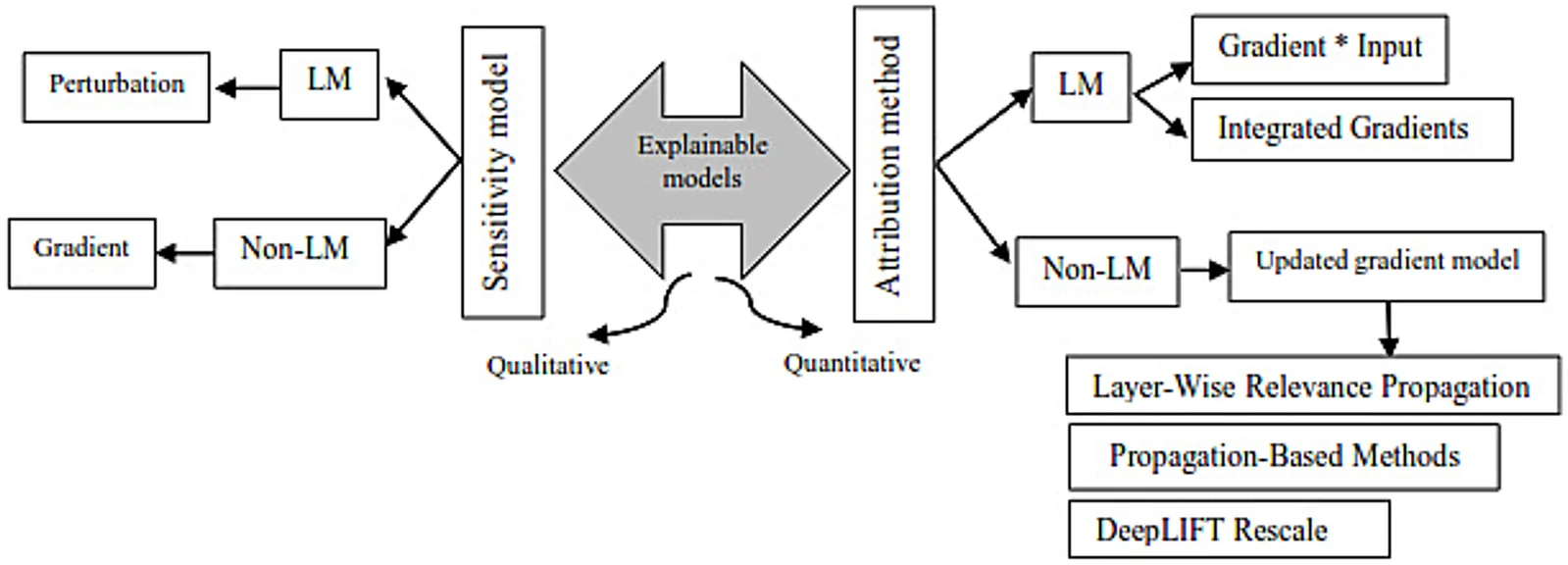
Taxonomy of gradient-based interpretability methods.

#### Sensitivity methods

Sensitivity methods examine the reaction of the model to small perturbations in the input and mainly provide a qualitative interpretation of the model’s behavior [53]. Two common methods in this category are saliency maps and feature removal.

One of the most widely used gradient-based techniques is the saliency map. In this method, the gradient of the output with respect to the input is computed, and the magnitude of this gradient shows how much each feature affects the model’s prediction. This method is simple, fast, and applicable to complex neural networks such as convolutional neural network (CNNs), recurrent neural networks (RNNs), and Transformers. However, it is sensitive to noise and only reflects the local behavior of the model. Saliency maps usually provide a qualitative analysis. The general formula is:


(2) \begin{eqnarray*} Saliency\left({x}_i\right)=\ \mid \frac{\partial f(x)}{\partial{x}_i}\mid \end{eqnarray*}

In contrast, a subset of sensitivity methods, known as feature removal, aims to measure the contribution of each feature by removing or deactivating that feature. In this method, all features are kept fixed and only one feature is removed at a time so that its effect on the model output can be measured. This process is repeated for all features to determine their relative importance. In nonlinear models, interpreting this method is more difficult due to the complex structure of the model.

#### Attribution methods

Attribution methods, instead of only examining local behavior, aim to assign a quantitative importance score to each input feature and usually provide more stable and reliable results. $\mathrm{Gradient}\times \mathrm{Input}$, IG (Integrated Gradients), DeepLIFT, and Layer-wise Relevance Propagation (LRP) are among the methods in this category [53].

In the Gradient × Input method, the attribution of a feature is computed by multiplying the feature value by its gradient. Compared to saliency maps, this approach provides a more quantitative and precise interpretation:


(3) \begin{eqnarray*} {Attribution}_i={x}_i\times \frac{\partial f}{\partial{x}_i} \end{eqnarray*}

The IG method addresses the problem of zero gradients in saturated regions by integrating the gradients along a path between a baseline input and the actual input. It is considered one of the most accurate attribution methods in deep networks and alleviates many limitations of raw gradients. In DeepLIFT, changes in the output are tracked with respect to a reference input in an additive manner. Compared to methods based on raw gradients, DeepLIFT is less sensitive to noise and saturation and usually provides more stable explanations. In LRP, the relevance of the model output is redistributed layer by layer back to the inputs. This method directly incorporates the network architecture into the computations and is very suitable for analyzing deep models with complex structures.

### Game-theoretic approach: Shapley value

Game theory analyzes the decisions or interactions of actors in an environment (economic, model-based, or other) using strategic mathematical models [56, 61]. In other words, it provides a framework for understanding how these players make decisions and predicting the consequences of their interactions, which can also be used as a method for interpretation in machine learning models.

Lloyd Shapley introduced the Shapley value. This value is a key concept in cooperative game theory that distributes the total payoff among players [55, 56]. The Shapley value computes each player’s marginal contribution to every possible coalition in the game and averages these contributions across all possible permutations. In this way, each player’s contribution is identified and rewarded appropriately. The Shapley value presents an unbiased distribution of the benefits of cooperation among players in cooperative games. It is widely used in fields such as economics, political science, computer science, and bioinformatics.


(4) \begin{eqnarray*} {\varnothing}_{\mathrm{i}}=\sum_{\mathrm{S}\subseteq \mathrm{N}\backslash \left\{\mathrm{i}\right\}}\frac{\left|\mathrm{S}\right|!\left(\mathrm{n}-\left|\mathrm{S}\right|-1\right)!}{\mathrm{n}!}\left[\mathrm{v}\left(\mathrm{S}\bigcup \left\{\mathrm{i}\right\}\right)-\mathrm{v}\left(\mathrm{S}\right)\right] \end{eqnarray*}

The symbol ${\varnothing}_{\boldsymbol{i}}$ in Equation (4) denotes the Shapley value assigned to player *i*. The variable *N* is the set of all players, and *v(S)* represents the value attributed to coalition *S*. The term |S| shows the number of players in coalition *S*. This summation considers all subsets *S* of *N* that exclude player *i*, thereby providing a comprehensive evaluation of player ${i}^{{\prime}}$s contribution across different coalitions.

### Transformers

To help readers with a bioinformatics background better follow the discussion, we briefly define here the key NLP and transformer concepts used throughout this paper in both NLP and bioinformatics. In transformer models, each small unit of input data is called a token. For example, in genomics, these units can be *k*-mers (or nucleotides), and in protein sequences, they are residues. Tokenization means converting raw inputs (such as DNA sequences or protein chains) into these tokens. The transformer model then processes these tokens to identify complex structural or functional patterns in the data.

Transformers have been introduced as a highly effective architecture for processing sequential data in NLP. This architecture (Fig. 7) was published by Vaswani *et al.* in the 2017 study “Attention is All You Need” [49]. Transformers have since become the base of numerous state-of-the-art NLP models. The main innovation in transformers is the attention mechanism, which focuses on different parts of the input sequence to predict the output. This mechanism enables transformers to get long-range relationships in text. Transformers are highly effective and powerful for tasks such as language translation, sentiment analysis, text generation, classification in bioinformatics, predicting binding sites, and more. Transformers have significantly advanced the capabilities of NLP and bioinformatics models. Pretraining large transformer models is rich because they learn from vast amounts of data and expand contextually sensitive language representations. Finally, these pretrained models can be fine-tuned on smaller, task-specific datasets for various NLP tasks, including text classification, named entity recognition, and question-answering.

**Figure 7:**
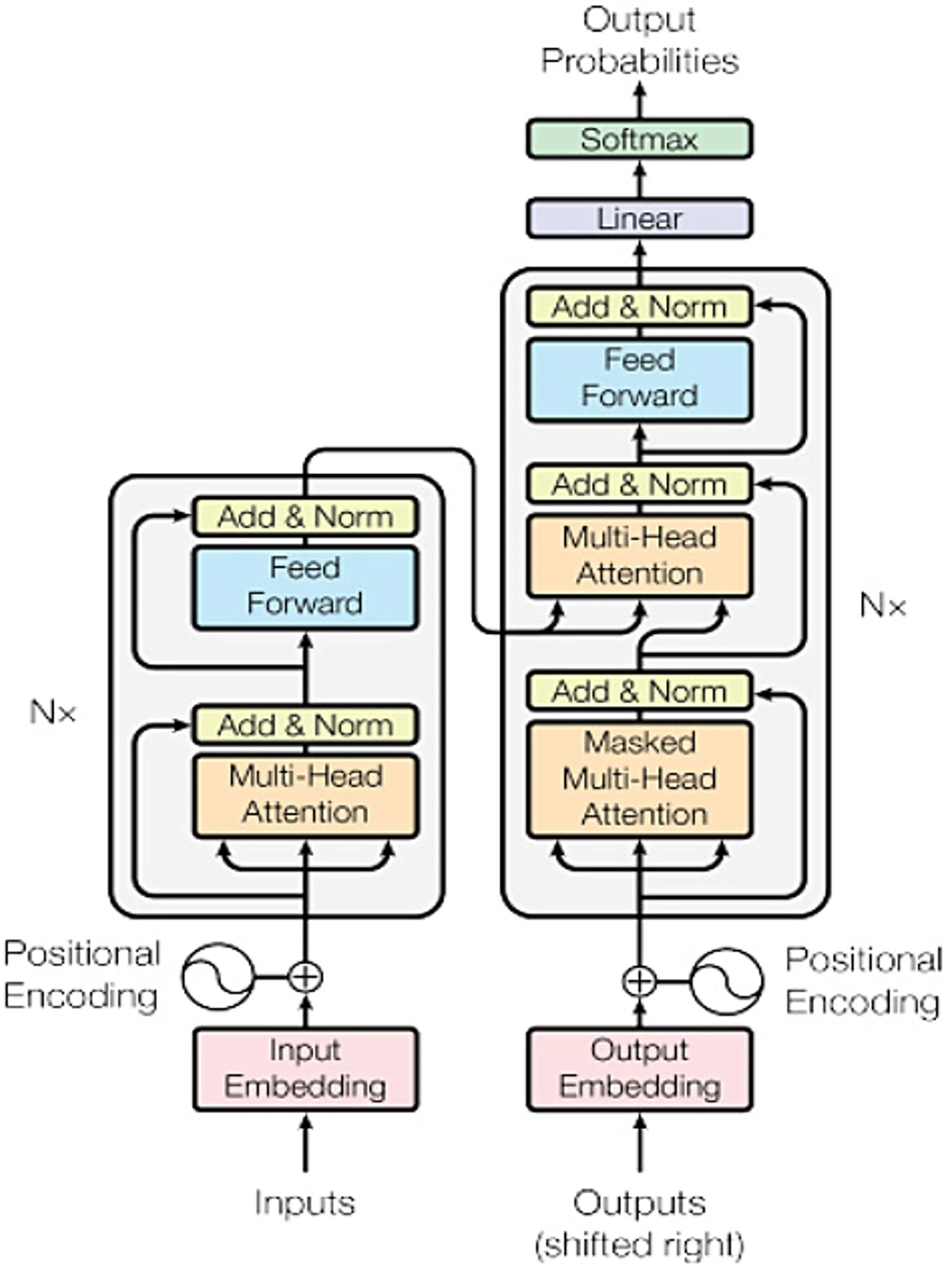
The transformer architecture [49].

After the development of transformer models in NLP, these architectures were extended to solve various problems in bioinformatics. For example, ESM (Evolutionary scale modeling) is a transformer-based protein language model trained on large-scale protein sequences to learn biological representations useful for downstream tasks. Similarly, AlphaFold employs attention-based deep learning architectures to predict the 3D structure of proteins with remarkable accuracy [62]. These models have demonstrated the significant potential of transformer architectures in bioinformatics applications.

In continuation, we examine various components of the transformer [49]:

#### The attention mechanism in transformer models

In transformers, using scaled Dot-Product attention (Fig. 8), the Query matrix is first multiplied by the Key matrix. This product is then scaled by $\frac{1}{\sqrt{d_k}}$​ to stabilize the gradients during training. Subsequently, a mask is applied to control the specific positions of interest. Finally, softmax is used to obtain the attention weights. These weights are then multiplied by the Value matrix, resulting in the final output of the attention mechanism.


(5) \begin{eqnarray*} Attention\left(Q,K,V\right)= sofrmax\left(\frac{QK^T}{\sqrt{d_k}}\right)V \end{eqnarray*}

**Figure 8:**
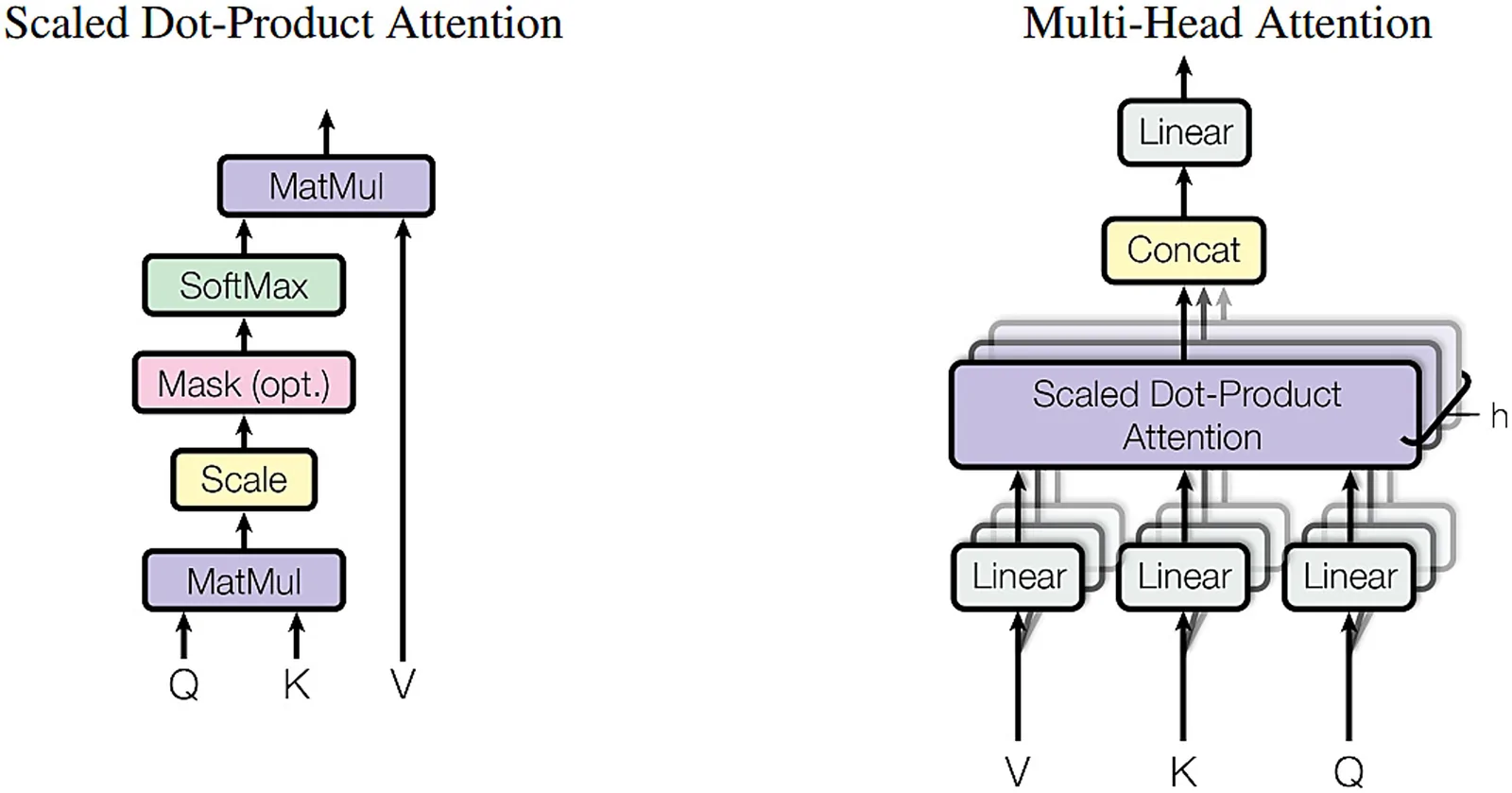
The Scaled Dot-Product Attention mechanism. Q, K, and V denote the Query, Key, and Value matrices, respectively. Adapted from [49].

In multi-head attention (Fig. 8), the operations performed in Scaled Dot-Product Attention are executed in parallel *h* times. The variable *h* represents the number of attention heads. Each head is applied independently to the input data. The heads’ results are concatenated to combine diverse features from the input. The role of multi-head attention in explainability is very prominent, as many analysis methods examine the importance of each head, eliminate heads, or aggregate attention. Attention weights are usually one of the most important sources of information in explainable methods and are used to analyze dependencies, the most important heads, or information paths.


(6) \begin{eqnarray*} MultiHead\left(Q,K,V\right)= Concat\left({head_1},\dots{},{head}_h\right){W}^o \end{eqnarray*}

$$where\ {head}_i= Attention\left(Q{W_i^Q},K{W_i^K},V{W}_i^V\right)$$

#### Positional encoding

Recurrence and convolution preserve the order of tokens during processing, but Transformers do not utilize them. Instead, to preserve the order of tokens, position along with the tokens is employed. For this purpose, sine and cosine functions express the positional information of the tokens.


(7) \begin{eqnarray*} PE\left({pos},2i\right)=\sin \left(\frac{pos}{10000^{\frac{2i}{d_{model}}}}\right) \end{eqnarray*}

$$PE\left({pos},2i+1\right)=\cos \left(\frac{pos}{10000^{\frac{2i}{d_{model}}}}\right)$$

In Transformers, tokens can be processed in parallel because they are not processed sequentially, leading to increased processing speed. The effect of order is significant in interpretive analyzes, especially in biological models (e.g. amino acid sequences).

#### Position-wise feed-forward networks

A sub-layer called a position-wise feed-forward network is embedded in the encoder and decoder layers of the Transformer. Unlike traditional feed-forward methods, this method processes tokens based on their positions. Each feed-forward layer consists of two linear transformations followed by a nonlinear activation function (ReLU).


(8) \begin{eqnarray*} FFN(x)=\max \left({0},x{W}_1+{b}_1\right){W}_2+{b}_2 \end{eqnarray*}

The input and output dimensions in position-wise feed-forward networks are 512, and the dimensions in the intermediate layers are considered 2048. One widely used transformer-based model is BERT (Bidirectional Encoder Representations from Transformers), a benchmark for many interpretability methods. Consequently, we provide a concise overview of BERT.

## Methods

This section presents a comprehensive classification of explainable and interpretable transformer methods. Some comprehensive methods for interpreting different models have been provided for researchers as tools for interpreting deep learning models, especially transformers. In the second section, these tools will be introduced.

### Classifying explainable transformer methodologies

Explainability is essential in various domains and models, including computer vision, NLP, and bioinformatics. In this review, we aim to explore the interpretability of transformer-based models on NLP and bioinformatics, as these two domains significantly overlap in modeling and processing [41]. Also, many models developed in NLP have been adapted and extended for use in bioinformatics. One of the most prominent models in both areas is the BERT model [63]; according to Table 2, many explainability techniques have been developed to explain the inner workings of the BERT model. Since the advent of BERT, several adaptations and advancements, such as RoBERTa (Robustly optimized BERT pretraining approach) [64] and DistilBERT (A distilled version of BERT) [65], have emerged, each addressing particular aspects of the original BERT architecture.

**Table 2:** Taxonomy of interpretability approaches for transformers in NLP and bioinformatics.

Paper	Field	Analyzed models	L/G	P/I	A/S	N/W
[73]	NLP	BERT	L	P	S	W
[76]	NLP	All models	L	I	A	W
[77]	Bio	ProtT5 + HyperGMA	L	I	S	W
[79]	Bio	Clinical Transformer	G	P	S	W
[54]	NLP	BERT, RoBERTa, XLNET	L	P	A	W
[66]	Bio	Sparse transformer	G	P	S	W
[67]	NLP	Bert	L	P	S	W
[80]	Bio	Transformer-XL+ Convolutional layer	L&G	P	S	W
[82]	NLP	Bert, DistillBERT, GPT-2	L&G	P	S	W
[68]	NLP	Bert	L	P	S	W
[57]	NLP	Bert, Transformer-based NMT	L&G	I	S	N

L, local; G, global; P, *post hoc*; I, intrinsic; A, agnostic; S, specific; N, norm-based analysis; W, weighted-based analysis

In this review, we strive to provide a classification of explainability methods for transformer models in both NLP and bioinformatics. As illustrated in Fig. 9, these methods can be categorized into two primary groups: weight-based methods and norm-based methods. Within the weight-based category, we have classified existing methods into five subcategories (gradient-based analysis, probing-based analysis, self-explaining, LIME-based analysis, and attention-based analysis) and will analyze each in depth.

**Figure 9:**
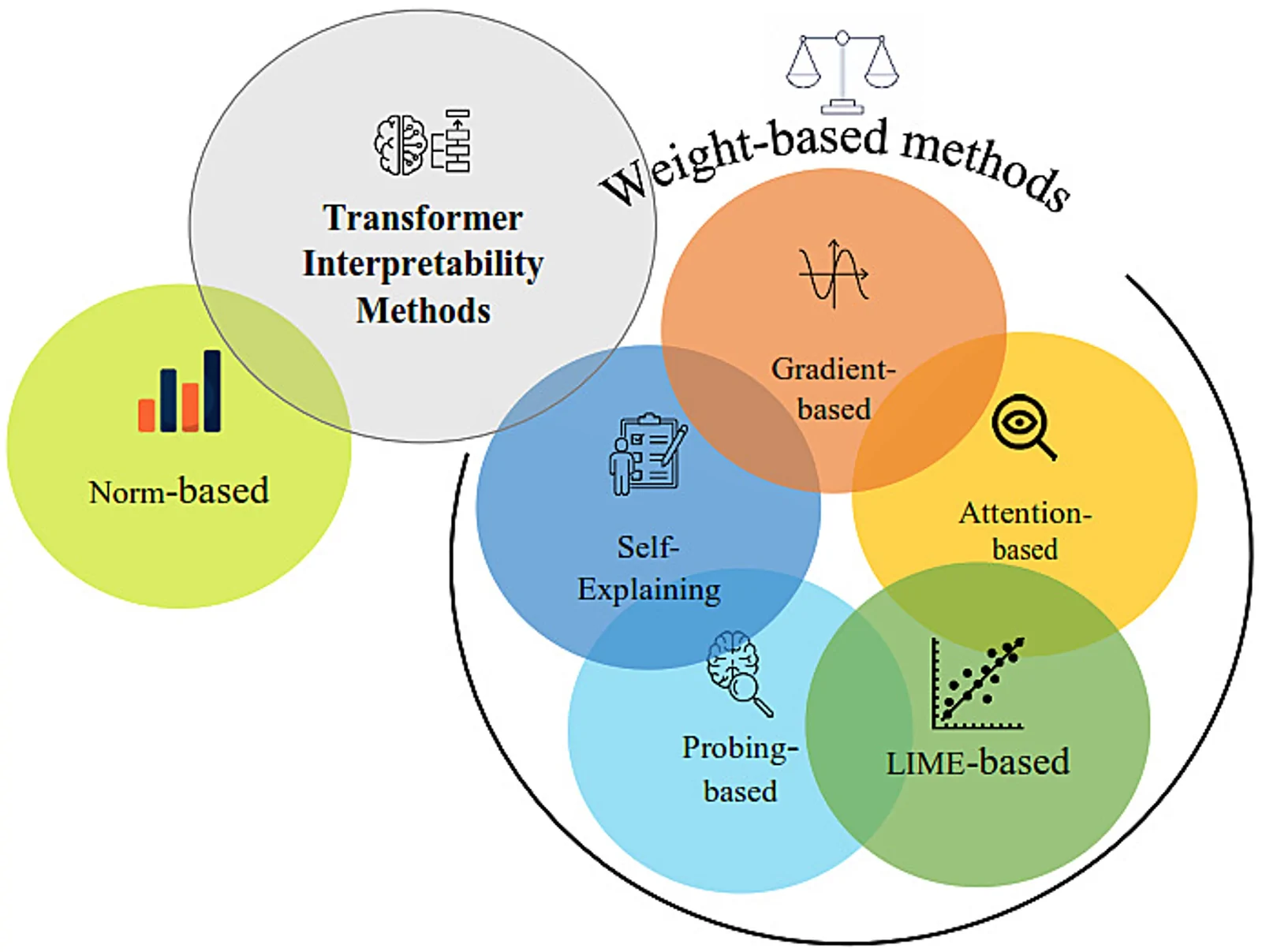
Explainability methods of transformers in NLP and bioinformatics, grouped into norm-based and weight-based families.

#### Methods based on gradient in transformers

This article [66] investigates the relationship between single nucleotide polymorphisms (SNPs) and diseases using a sparse transformer model (Fig. 10). According to numerous genome-wide association studies (GWAS) studies, SNP–disease associations have been highlighted as a significant challenge in biomedicine and personalized healthcare. Many genetic diseases are explained by the interactions of multiple SNPs, known as epistasis. Therefore, an interpretable model is strongly needed to predict these complex interactions. Given the crucial importance of interpretability in the biomedical field, the authors of [66] proposed a modified transformer method that uses sparser matrices at different levels to identify and analyze epistasis with greater flexibility. The proposed method integrates dimensionality reduction techniques (PCA (Principal component analysis), LLE (Locally linear embedding), SE (Spectral embedding)) with a Top-KAST (Top-K always sparse training) gradient-based strategy, in which only the top K gradient weights are updated during training (Fig. 10b). The performance of the presented method is enhanced while preserving the model’s ability to identify key SNP interactions through a subsequent exhaustive search based on attention scores (Fig. 10a), thereby balancing interpretability and accuracy in biomedical applications.

**Figure 10:**
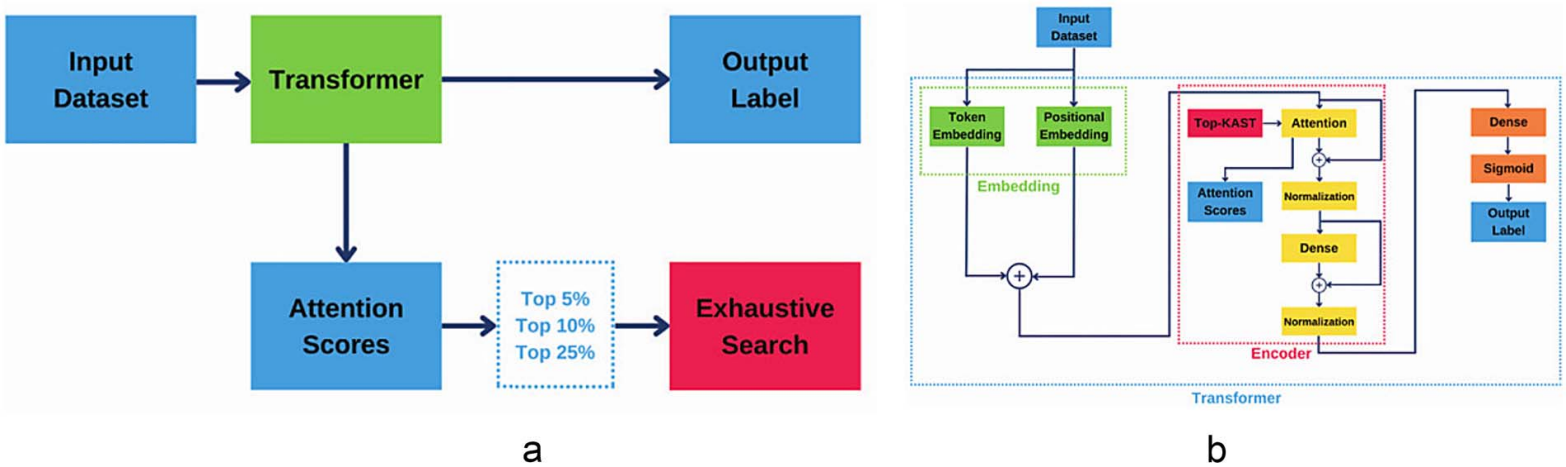
The sparse transformer architecture for epistasis detection [66]. (a) The overall framework showing how attention scores guide the exhaustive search for SNP interactions. (b) The internal encoder structure incorporating the Top-KAST strategy to update only the most significant gradients. a) The Framework or Epistasis Detection. b) Transformer Architecture with Top-KAST strategy.

In another interpretability study in NLP, the study introduces a gradient-based interpretability method to measure paragraph similarity using the BERT model [67] (Fig. 11). The features of each paragraph are encoded as embedding vectors, and cosine similarity is used to quantify their relationship. Then, the gradients of one paragraph relative to another are calculated to determine how each feature contributes to the similarity. Finally, salience scores are calculated based on gradient values to highlight features and enable the discovery of related keywords through clustering.

**Figure 11:**
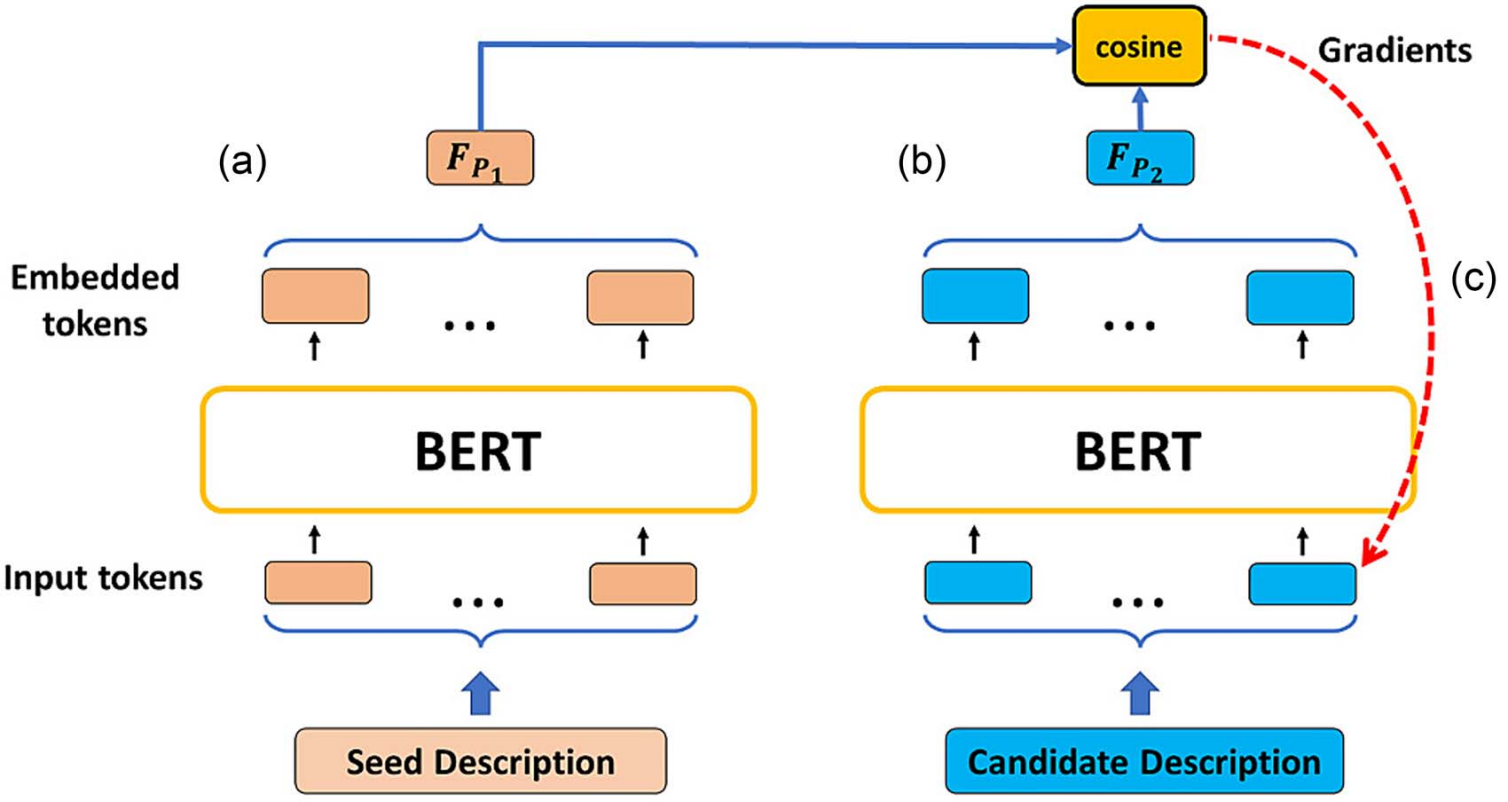
Interpreting the similarity between two paragraphs using the BERT model [67]. (a) and (b) Independent BERT encoders generate feature embeddings for the seed and candidate descriptions. (c) Gradients flow through the cosine similarity layer, indicating how individual tokens influence the similarity score.

As shown in Fig. 11, two separate BERT encoders (A and B) process the seed and candidate paragraphs. The cosine similarity module compares their feature representations, and the red dashed arrow (C) illustrates how the gradient flows backward from the similarity score to identify influential tokens in each paragraph. This visualization clarifies how feature importance and interpretability are derived from cross-paragraph gradient interactions.

Du and colleagues [68] extended the concept of gradients using semi-loop-based back propagation. They introduced (max, ×) and entropy gradients to analyze information flow through attention and skip connections in transformers. Two new gradient formulations are introduced: the (max, ×) gradient, which tracks the dominant gradient flow along the strongest path (the top gradient path), and the entropy gradient, which captures the overall dispersion of gradients across multiple paths. This method has been tested on the transformer model using the (max, ×) gradient. In accordance with the test findings, the proposed approach effectively recognizes the interactions between key tokens in self-attention. However, the entropy gradient provides a broad view of the network’s information flow and increases the interpretability of the transformer’s internal mechanisms.

While gradient-based methods provide useful insights into how input features influence model predictions, an important methodological question concerns the faithfulness of such explanations. In many cases, gradient-based saliency scores highlight features that correlate with the model output, but they do not necessarily reveal the true causal mechanisms underlying the model’s decision process [69, 70]. Previous studies have shown that gradient-based explanations can sometimes be unstable or sensitive to small perturbations in the input or model parameters, which raises concerns about their reliability as faithful explanations [71]. In addition, gradients primarily capture local sensitivity around a specific input rather than the global behavior of the model [72]. As a result, the highlighted tokens or features may represent plausible indicators of importance rather than the actual reasoning process used by the model.

Furthermore, the interpretation of gradients can be affected by architectural properties of transformer models, such as deep layer structures, residual connections, and attention interactions, which complicate the propagation of attribution signals through the network [71]. These factors may cause gradients to reflect indirect or diffuse contributions rather than clearly identifiable causal pathways. In practice, gradient-based interpretation may be affected by several factors such as model structure, data noise, and feature range. These factors can lead to biased interpretations with low accuracy [68]. Consequently, although gradient-based approaches are computationally efficient and easy to apply, their explanations should be interpreted with caution and ideally complemented with other interpretability techniques, such as perturbation-based analysis or mechanistic interpretability methods, to obtain more robust insights into transformer decision-making processes.

#### Methods based on probing attention head

Probing methods are analytical techniques that assess which linguistic or structural information is encoded in the representations of pretrained models by training simple auxiliary classifiers on internal representations, such as hidden states or attention patterns. Studies on BERT show that different attention heads detect specific behaviors [73]. Some heads focus on fixed positional tokens, while others distribute attention widely in the input sequence. Systematic attention to structural tokens such as [SEP] indicates that some heads capture delimiter or structural information rather than purely syntactic relations. Figure 12 further shows that heads within the same layer often exhibit similar behaviors, while several heads specialize in attending strongly to the previous or next token, particularly in earlier layers of the model. These analyses suggest that attention heads capture positional, structural, and syntactic patterns that contribute to the model’s predictions.

**Figure 12:**
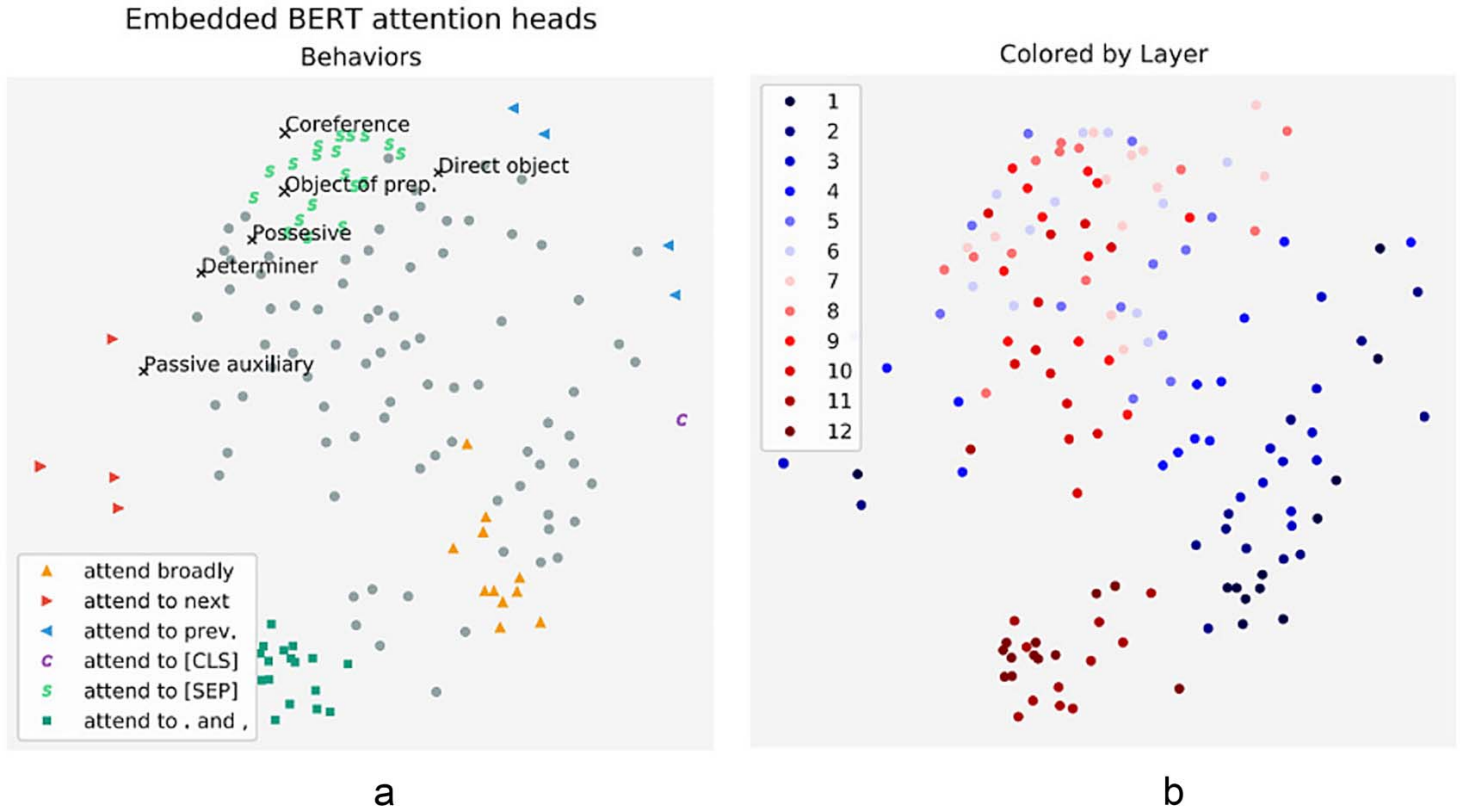
A 2D embedding of BERT attention heads based on similarity of attention patterns (measured using Jensen–Shannon divergence). Each point represents an attention head, with markers grouped and labeled by layer (1–12). Heads from the same layer exhibit similar patterns and tend to cluster together. Several early-layer heads attend strongly to the previous or next token [73]. (a) Analysis of head behaviors. (b) Analysis of layer-wise patterns.

Probing attention heads enables the discovery of latent syntactic and positional patterns encoded in transformer models. However, not all attention heads exhibit interpretable behavior, and probing results may be influenced by external classifiers or embedding biases, limiting the faithfulness of the explanations.

##### Mechanistic interpretability and causal circuit analysis

Recent studies in model interpretability have introduced an approach known as mechanistic interpretability for analyzing transformer models. Unlike many traditional interpretability methods that operate in a *post hoc* manner and mainly examine correlations between model outputs and input features, mechanistic interpretability focuses on identifying the internal computational mechanisms that give rise to a model’s behavior. In this perspective, the goal is essentially to reverse-engineer transformer models by analyzing how different components—such as attention heads, neurons, and multilayer perceptron (MLP) blocks—interact with one another to perform specific algorithmic computations.

One influential framework in this area is the transformer circuits approach proposed by Elhage [74]. This framework analyzes how information flows through attention heads and feed-forward layers and how these components together form computational circuits within the model. These circuits can carry out relatively interpretable operations, such as copying tokens, detecting patterns, or aggregating contextual information. Subsequent studies have also identified specific structures within transformers, such as induction heads, which enable models to perform a form of in-context learning by recognizing and repeating patterns within sequences [75]. These findings suggest that transformer models may develop reusable algorithmic components, where different layers and attention heads collaborate to implement specific computational functions.

A key distinction between mechanistic interpretability and traditional approaches such as probing or attribution methods is that mechanistic interpretability seeks causal explanations rather than merely descriptive correlations. Instead of asking whether a model representation encodes a particular feature, this approach attempts to determine which internal components actively carry out the computations that lead to the model’s prediction. Although most current studies focus on relatively small models due to the difficulty of analyzing very large networks, mechanistic interpretability represents a promising research direction for achieving deeper and more reliable explanations of transformer-based systems.

#### Self-explaining methods

One prominent deep approach to interpreting transformer architectures is the self-explaining method. Unlike *post hoc* interpretability techniques, this approach integrates the explanatory mechanism directly into the model and refines it throughout training. As argued in [76], self-explaining models can provide a deeper understanding of their own decision-making processes because the explanatory components monitor how predictions are formed internally. In this sense, self-explanation modules should not be viewed as external diagnostic tools but as integral parts of the model itself [76] (Fig. 13). Figure 13 presents the internal pipeline of the self-explaining model, where the interpretation layer computes explanatory coefficients ($\alpha \left(i,j\right)$) from intermediate representations (${h}_1-{h}_5$), and the SIC (Span Infor collecting) layer aggregates these signals into the final output (Positive). Earlier research has outlined several motivations for self-explainability. First, many interpretable models rely on an additional surrogate model to generate explanations, which often leads to approximate or potentially unfaithful interpretations [76]. Second, most *post hoc* approaches operate at the word level, making it difficult to extend explanations to broader textual units such as phrases, sentences, or paragraphs [76]. By embedding interpretability within the model’s architecture, self-explanation methods address these shortcomings and produce explanations aligned with the model’s internal reasoning. They facilitate error analysis, assist downstream NLP tasks such as adversarial example generation, promote architectural transparency, and, in some cases, even enhance overall performance [76].

**Figure 13:**
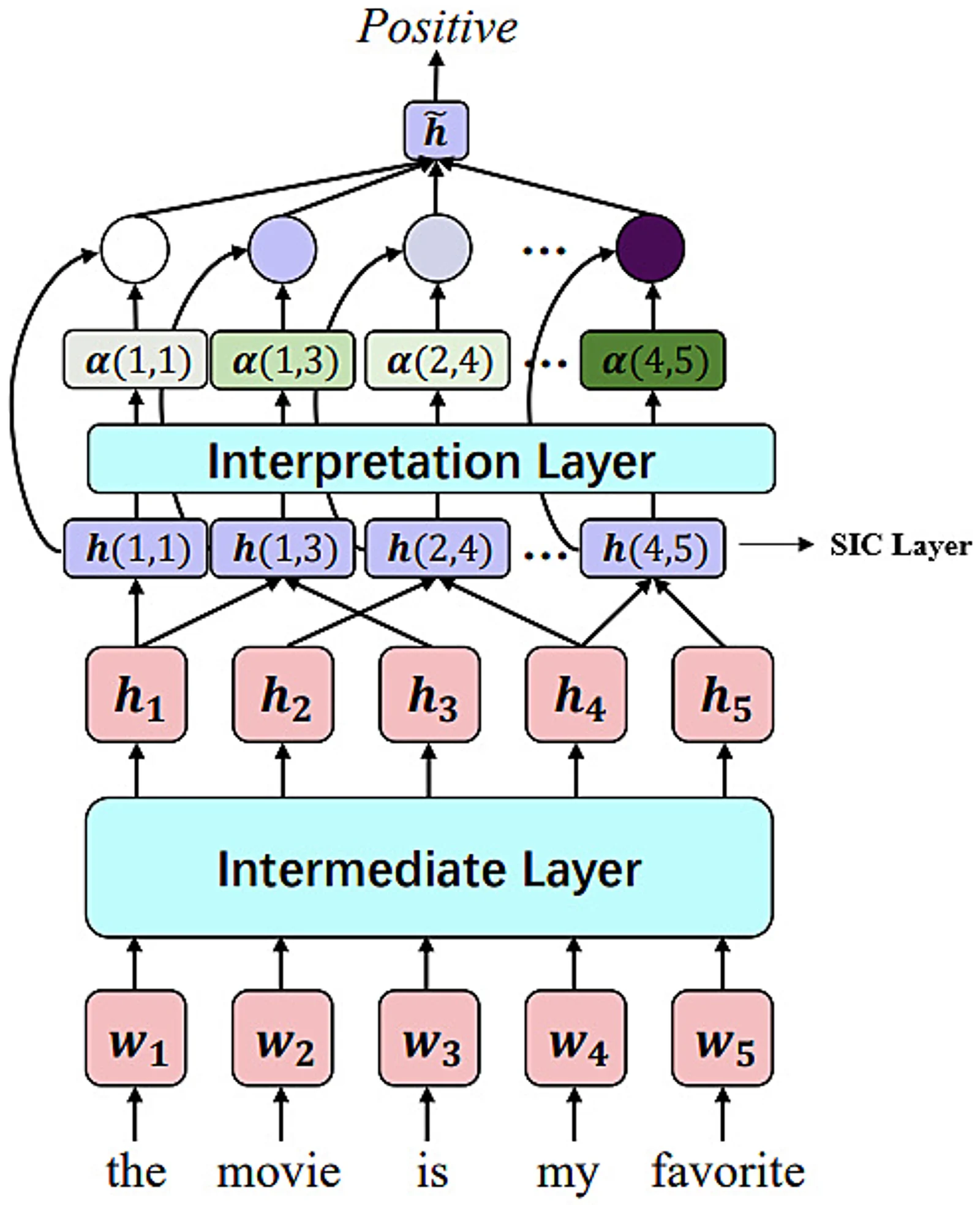
Overview of a self-explaining framework in which prediction and explanation components are jointly learned, enabling interpretability to emerge directly from the model’s internal representations [76].

Beyond general NLP tasks, self-explaining methods have also demonstrated strong potential in biological sequence analysis. The PHAT (Hypergraph Learning and Transfer Learning based Framework for Peptide Structure Exploration) [77] framework presents an innovative methodology for predicting peptide secondary structure while maintaining interpretability (Fig. 14). Fundamentally, PHAT consists of three main phases. In the first phase, knowledge is transferred from a pretrained ProtT5 model trained on the UniRef50 dataset. Through self-supervised learning, this stage enables knowledge transfer from proteins to peptides. In the second phase, peptide embedding vectors are refined using a hypergraph model. In the final phase, the outputs of the previous stages are integrated, and a Bi-LSTM layer is employed to enhance sequence representation. The output of the Bi-LSTM is then passed to a CRF (Conditional Random Fields) layer to predict the peptide’s secondary structure. Within the CRF layer, the model learns two key matrices—transition and emission—that help explain the prediction mechanism of PHAT. CRFs are probabilistic graphical models widely used for sequence-labeling tasks such as secondary-structure prediction. The emission and transition matrices model the probabilities of structural states and the transitions between them, thereby providing insight into how the model generates its predictions.

**Figure 14:**
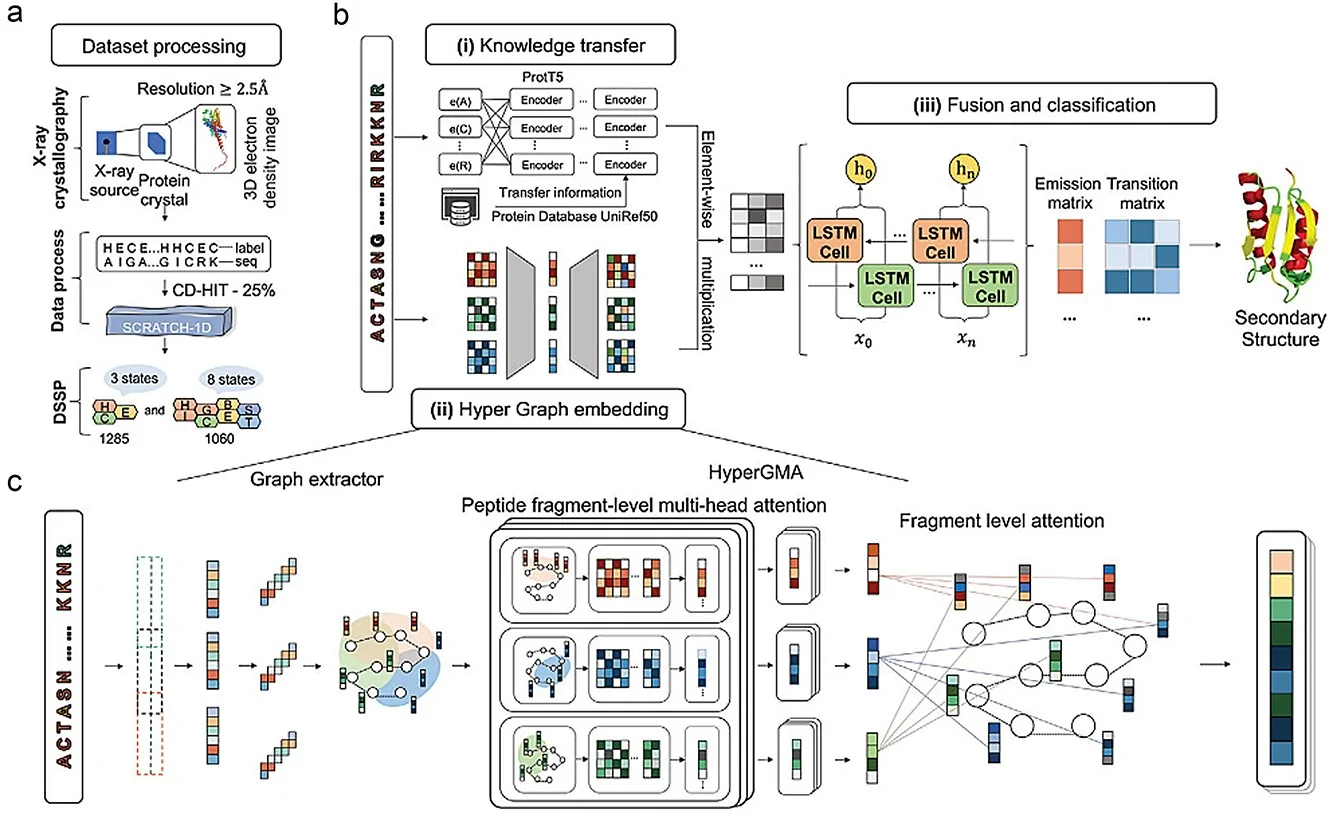
Overview of the PHAT framework for peptide secondary structure prediction, illustrating knowledge transfer, hypergraph embedding, and BiLSTM-CRF-based classification [77].

Self-explaining models have also gained attention in cancer research, where interpretability is essential for clinical trust and decision-making. In the review [78], 42 articles on cancer omics with an emphasis on model interpretability were analyzed. Various architectures, including CNNs, VAEs, sparse VAEs, transformers, and other deep learning models, were examined. This review study placed significant emphasis on model interpretability and also critiqued several interpretation approaches.

A study [79] presents a clinical transformer framework for modeling the complex interactions between molecular and clinical characteristics and their implications for patient survival outcomes. One of the major challenges in cancer research is its multifaceted and heterogeneous nature across individuals. Researchers aim to harness the potential of immunotherapy in cancer treatment and its ability to control the patient’s immune system to fight the disease. In this research, the researchers aim to discover meaningful correlations between variables related to the patient’s physical condition, immune status, and tumor characteristics in order to analyze the fundamental mechanisms of immunotherapy. This approach seeks to uncover latent clinical phenomena associated with immunotherapy and to provide insights into therapeutic mechanisms that influence patient responses. In this study, both molecular and clinical features are used as model inputs to predict survival outcomes. To enhance interpretability, the VIS metric was introduced to rank features based on aggregated attention scores, enabling clinicians to examine how molecular and clinical variables jointly contribute to survival predictions.

In Article [80], an interpretable method is presented to understand the transcription process in *Escherichia coli*. The model proposed in this research is a modified version of the Transformer-XL [81] model. In addition to using attention mechanisms, it employs convolutional layers to capture local information between nucleotides. Analysis of attention heads revealed biologically meaningful patterns, such as those focused on transcription factor binding regions and conserved motifs, such as the TATAAT sequence. This discovery helps identify factors that influence transcription initiation. These findings show that self-explaining architectures can naturally learn and highlight biologically relevant signals directly from sequence data without the need for external interpretation tools.

In general, self-explanatory methods are integrated into the model architecture and therefore better align with the model’s actual decision-making process. These models can highlight semantic patterns and relationships in data and enable interpretable learning, especially in complex domains such as biomedical and clinical applications [78–80]. However, self-explanatory methods often require modifications to the underlying model structure and are therefore not easily applied to pretrained models. In addition, some models have complex, multi-stage architectures that may make implementation and training more difficult [77].

Another important methodological consideration concerns the faithfulness of the explanations generated by these models. Although self-explanatory mechanisms are embedded within the architecture and are therefore more closely aligned with the model’s internal representations, the explanatory signals they produce may still reflect correlations rather than the true causal reasoning process of the model. For example, explanations derived from attention mechanisms or aggregated attention scores may highlight influential tokens or features without necessarily establishing a direct causal relationship with the model’s prediction.

Furthermore, complex architectural components such as multi-layer attention interactions, residual connections, and embedding representations may influence how explanatory signals propagate through the network. As a result, the highlighted patterns should be interpreted carefully and ideally supported by qualitative analysis or domain expertise, particularly in sensitive applications such as biomedical and clinical decision support. Consequently, although self-explanatory models provide a promising direction for integrating interpretability directly into model design, human evaluation and domain-specific validation remain essential to ensure the reliability and usefulness of the generated explanations.

#### Methods based on LIME

LIME is designed to explain model predictions using an interpretable linear approximation. Several perturbed samples are generated around each original data point, then a simple linear model is trained on them. The influential features in the projections are identified using the weights of this linear model. This method has applications in model interpretation and stability analysis. Model stability refers to a model’s ability to exhibit little change in output in response to minor perturbations. In reference [54], three transformer-based models in the NLP domain (BERT, RoBERTa, XLNet) are subjected to adversarial attacks. Adversarial attacks involve generating data that misleads the model. A model that makes fewer mistakes in response to these attacks is more stable. The results show that the BERT model is most susceptible to adversarial attacks. In the following, we will examine the advantages and limitations of this method.

The LIME method explains the prediction of each sample separately. This characteristic is particularly useful in areas such as NLP and bioinformatics, as it facilitates a better understanding of the decisions made by complex models. Another advantage of the LIME method is its simplicity and flexibility. It approximates the model’s behavior near the sample using a linear surrogate that is easy to interpret. The LIME method is also used to assess the model’s stability and resistance to minor changes.

In addition to its advantages, the LIME method has several limitations. The first challenge of this method is that it is only valid in the local area and may not accurately reflect the model’s overall behavior. The second limitation is LIME’s dependence on sampling and the number of samples. If the sampling is insufficient, the surrogate model will not accurately capture the behavior of the original model. This problem is exacerbated in high-dimensional spaces, such as those in NLP, because it is challenging to make small, meaningful changes to text or sequences, which may result in unnatural or meaningless alterations. Another point is that the LIME method can be unstable in interpretation because it relies on random sampling. If we repeat the process several times, we may obtain different results, which reduces confidence in the interpretation result, especially in sensitive areas such as medicine and biomedicine. Another important point is that the LIME method does not consider the interaction of features. In models such as transformers, whose mechanisms depend heavily on feature interactions, this may lead to incomplete interpretations. Moreover, the LIME method faces a final challenge: it can only detect adversarial attacks and cannot improve the model’s stability.

#### Attention-based interpretability methods

Jain and Wallace [70] argue that raw attention weights alone are unreliable descriptors. Several studies have also questioned whether attention weights constitute faithful explanations of model predictions, suggesting that they may act only as plausible indicators of importance rather than causal explanations [70, 82]. These merely represent relative attention scores for inputs and do not allow direct comparisons between tokens. To overcome this limitation, two methods—attention rollout and attention flow—have been proposed to aggregate attention across layers (Fig. 15). Results show that these two methods, especially attention flow, have higher correlations (based on Spearman’s rank correlation) with gradient-based interpretability and input-elimination methods than raw attention [82]. Furthermore, at higher layers, attention patterns appear more transparent and more stable in the rollout and flow methods than in raw attention. In particular, attention flow, which models attention spread along with residual connections, provides more reliable and meaningful interpretations and has been shown to correspond to Shapley-value explanations of model predictions [83]. Overall, this study suggests that structured aggregation of attention across layers can yield more accurate interpretations than using raw attention weights alone.

**Figure 15:**
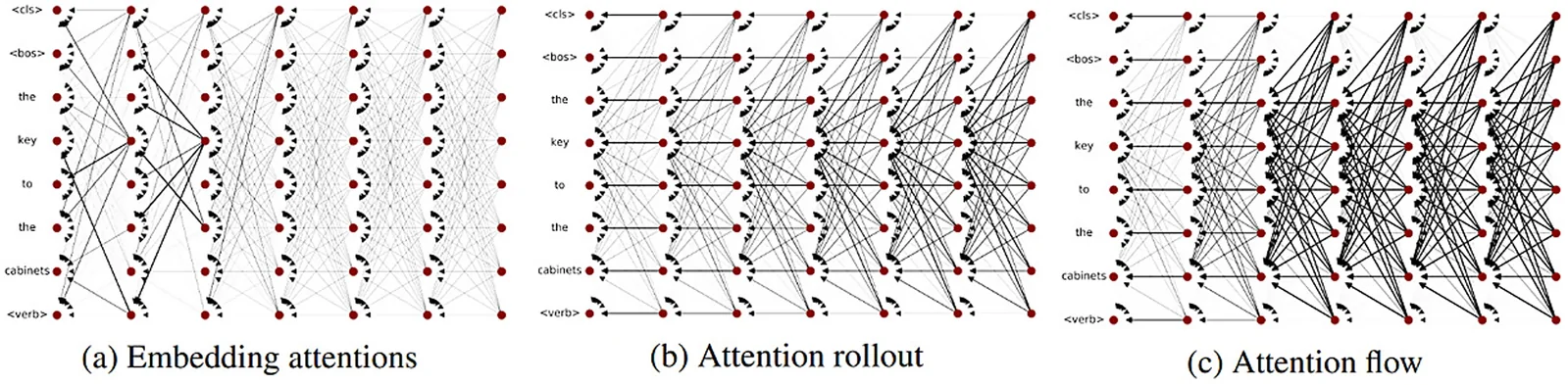
Comparison of attention visualization methods including raw attention (embedding attention), attention rollout, and attention flow. Raw attention represents layer-specific attention weights computed on the token embeddings, while rollout and flow aggregate attention across multiple layers to better capture how information propagates through the transformer architecture [82].

Despite these advantages, attention-based interpretability methods have certain limitations. The computational complexity of attention flow is high because it requires modeling a flow network [82]. Additionally, the attention rollout method relies on assumptions that may not hold for all models. Another challenge is that the residual connections in the models lead to misinterpretation, making it impossible to focus on the actual attention weight. However, as depth increases, the effect of residual connections diminishes, and the exact differences between interpretability methods become clearer [82].

#### Methods based on norm

The study in [57] introduces an interpretability method that focuses on two key factors: attention weights and the norms of the transformed input vectors. The authors argue that both factors must effectively contribute to the attention outputs for a faithful interpretation. A significant point emphasized in this work is that source–target attention should be analyzed to achieve logical alignment between words. Figure 16 presents an overview of the attention mechanism, where part (a) illustrates the standard mechanism, and part (b) shows the reformulated view based on the norm. In this approach [57], the basic attention equations are rewritten, utilizing the sum of weighted transformed vectors. According to Fig. 16b, all inputs *x* are first mapped to *f(x)* under the transformation function*f*, then the final output ${y}_i$​ is computed as the sum of weighted vectors:


(9) \begin{eqnarray*} {y}_i=\sum_{j=1}^n{\alpha}_{i,j}f\left({x}_j\right) \end{eqnarray*}

**Figure 16:**
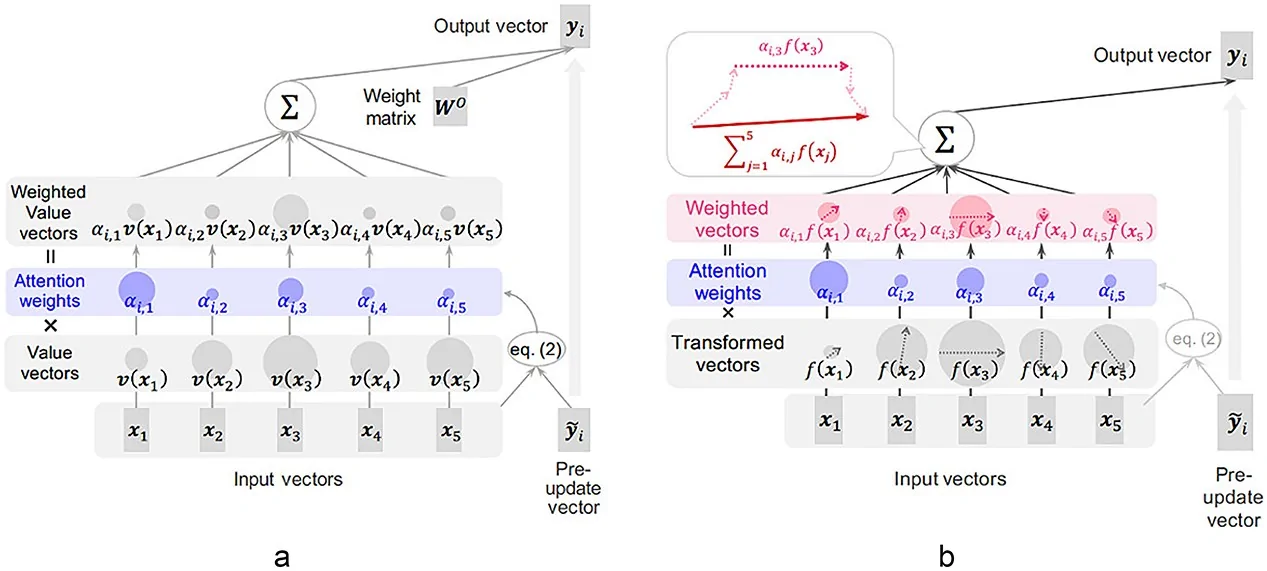
Overview of the attention mechanism: attention weights and norms of transformed input vectors [57]. (a) Overview of attention mechanism in Transformers. (b) Overview of attention mechanism based on ${y}_i=\sum_{j=1}^n{\alpha}_{i,j}f\left({x}_j\right).$

The primary motivation for using the norm is that in traditional weight-based methods, only attention weights (*α*) contribute to interpretation, disregarding the information embedded in the input vectors. As illustrated in Fig. 16b, the size of the circles representing the attention weights and the transformed vectors indicates their respective magnitudes; both components are influential in the final result. For example, a previous study [73], which focused solely on attention weights, concluded that special tokens like [SEP] and [CLS] receive disproportionately high attention. However, [57] demonstrates that when the norm of the transformed vectors $\mid \left|f(x)\right|\mid$ is included, these special tokens (along with punctuation like “.” and “,”) often show lower magnitudes, thereby reducing the perceived importance of these tokens and mitigating bias in the interpretation.

The Norm-based interpretability method offers several advantages, such as providing a more comprehensive analysis of the model’s performance by utilizing both weight and magnitude measures [57]. This method achieves better semantic alignment, where the final interpretation matches the semantic structure of the sentence more realistically. However, this method also presents challenges. It has higher computational complexity than attention-weight-only methods because it requires calculating and analyzing the transformed vectors [57]. Furthermore, because it requires reformulating the classical attention equations, it may be more difficult to implement, and the combined influence of weights and norms can make attention maps less intuitive for some users.

Another important methodological consideration is the faithfulness of the explanations. Although incorporating vector norms provides a more detailed view of the information flow, these signals may still represent correlations within the model rather than direct causal reasoning. The transformation function *f* can also introduce its own biases, meaning that highlighted patterns should be interpreted carefully. Therefore, while norm-based methods improve upon raw attention, human evaluation and domain-specific validation remain essential to ensure that the generated interpretations are truly reliable and useful in sensitive applications.

Table 2 shows a comprehensive overview of recent papers on the interpretability of transformer models in the two domains of NLP and bioinformatics. One of the highlights of this table is the widespread popularity of the BERT model and its derivatives in the field of NLP. Almost all studies in this area have directly or indirectly analyzed BERT-based structures, indicating the standardization of this model in interpretability assessments in NLP. In contrast, in the bioinformatics domain, despite the use of more diverse models, such as ProtT5, Clinical Transformer, and Sparse Transformer, there is a lesser focus on in-depth interpretability assessments. This architectural diversity, along with the fragmentation of interpretability approaches, suggests that bioinformatics is still in the early stages of standardizing interpretability methods. From the perspective of the four dimensions of interpretability (Local/Global, Post-Hoc/Intrinsic, Agnostic/Specific, Weighted/Norm), some important patterns are visible. Most papers focus on local and *post hoc* approaches, which represent an attempt to understand individual predictions without altering the original model’s structure. Most studies use weighted-based analysis, which seems natural, especially in attention-based models. The norm-based analysis is relatively rare and has received less attention. Generally, this table not only shows the most widely used models and approaches in the field of interpretability but also highlights gaps in the bioinformatics field—particularly the lack of structural or intrinsic methods for interpreting models. These shortcomings can guide future research toward developing interpretable tools that are compatible with the complex nature of biological data.

#### Comparative analysis of interpretability methods

In this section, we compare a set of interpretability methods that differ in terms of their dependence on model architecture, the need for labeling, the amount of user intervention, and the type of output (Table 3). The goal of this comparison is to highlight the properties and limitations of each approach and to examine how each technique can be effective in specific contexts, particularly in complex domains such as bioinformatics. For example, gradient- and attention-based methods are strongly dependent on the model structure but do not require labeling and can be used with pretrained models. These methods focus on the perception of the model’s internal behavior, but they can be sensitive to noise and may not reliably reflect the true importance of input features.

**Table 3:** Comparative overview of interpretability methods in transformer models.

Method	Architecture dependency	Label requirement	User involvement	Applicability in bioinformatics	Output type	Usable with pretrained models	Advantages	Limitations
**Gradient-based**	High	No	Medium	Yes	Heatmaps/saliency scores	Yes	High precision, captures internal model behavior	Sensitive to noise, unstable gradients
**Probing-based**	Low	Yes	High	Yes	Evaluation metrics/retained features	only auxiliary classifiers trained	Identifies influential linguistic/biological features via classification probes	Requires labeled data and careful probe design
**Self-explaining**	High	No	Low	Limited	Internal explanations (e.g. feature weights)	No (retraining required)	Built-in interpretability, direct insight into decisions	Needs specific model architecture, not widely adopted
**LIME-based**	Low	No	High	Yes	Local feature importance weights	Yes	Model-agnostic, interprets individual predictions	Computationally expensive, unstable for similar inputs
**Attention-based**	High	No	Low	Yes	Attention maps	Yes	Intuitive, easy to implement	Attention does not always correlate with importance
**Norm-based**	Medium	No	Low	Yes	Vector norms/visual scores	Yes	Combines input content and learned weights	Less conventional, may require additional interpretation strategies

In contrast, methods such as LIME and probing focus on external interaction with the model, i.e. they try to interpret the model by simply identifying the relationship between the input and output. LIME helps rank features by creating artificial data around the input and building a local linear model. At the same time, probing uncovers information hidden in pretrained models by designing simple models to predict specific information from embedding vectors. However, probing requires labeled data and careful probe design, and LIME is computationally expensive and sometimes unstable. Self-explaining models, although they have interpretation built directly into their architecture, usually require retraining and are not yet widely used.

On the other hand, norm-based methods, which use the size of latent vectors to infer importance, although not widely accepted, provide a bridge between the input content and the knowledge learned in the model. In general, all of these interpretability methods have strengths and weaknesses, and none alone addresses all of the user’s interpretability needs. Depending on the purpose of the analysis and interpretation, as well as the limitations of the model, each can play a key role. Therefore, the combined use of these methods can present a more comprehensive and robust approach to understanding the decisions of complex models.

The following table shows which categories of methods for interpreting transformer models each explainability tool supports. These categories include six main groups: Attention-based, Gradient-based, LIME, Probing-based, Norm-based, and Self-explaining models. A “✓” indicates that the tool supports the corresponding method. This table serves as a bridge between the practical tools and the theoretical foundations of explainability methods, enabling researchers to select the appropriate tool based on their desired method.

Table 4 can be analyzed from different perspectives. First, due to the alignment of the tools with the Transformer architecture, most of the tools focus on attention-based methods. Second, several tools, such as Language Interpretability Tool (LIT) and T-vis, offer a variety of interpretability methods. The variety of different interpretability methods is important for complex biomedical data. Some interpretability methods, such as Norm-based methods, have received very little attention despite having great potential for analyzing bioinformatics tasks.

**Table 4:** Correspondence between interpretability tools and explanation methods.

Tool	Attention	Gradient	LIME	Probing	Norm	Self -explaining
**Bertviz**	✓					
**EXBERT**	✓					
**Provis**	✓					
**T-vis**	✓	✓		✓		
**LIT**	✓	✓	✓	✓		
**Dodrio**	✓					
**AllenNLP**		✓				
**InterpreT**	✓					

In the following, we will examine each tool introduced in the table above separately. This review will cover the views, use cases, and key capabilities of the explainability methods, as well as their applications in various fields, particularly NLP and biology.

### Explainability tools for transformer models

Various frameworks, including [84–91], have been provided to interpret transformer models and to clarify their complexity and attention mechanism. Most of these frameworks focus on visualizing layer performance through attention heads, allowing researchers to observe how different layers behave. However, a deeper understanding of transformer performance is needed. Simultaneously, interpreting deep models, especially transformer models, can help generate new knowledge, generalize the deep model, increase the model’s accuracy, debugging, and optimize the model.

BertViz [84] is an interactive visualization tool for exploring attention patterns and internal representations in Transformer models. It supports three complementary views—the attention-head view, the model view, and the neuron view—allowing analysis at different levels of the architecture. Instead of relying on a single view, users can pivot between these levels to study how attention evolves—from raw token-to-token connections to the complex interactions happening across different heads and layers. The neuron view further allows inspection of query–key computations that shape the attention process. In real-world applications, researchers often use BertViz to isolate influential attention heads, detect hidden model biases, or correlate neuron activity with the model’s overall output. That said, it is worth remembering the authors’ own warning: attention patterns are useful, but they should be viewed as a diagnostic signal rather than a complete, definitive explanation for why a model makes a specific prediction.

One of the widely used tools for understanding the behavior of transformer models is the AllenNLP interpretability toolkit [85]. An overview of its components is shown in Fig. 17. It consists of three main components: Predictor, Interpreter, and Attacker. AllenNLP uses two main approaches: first, Saliency Maps (including Vanilla, Integrated Gradients, and SmoothGrad), which highlight the words that the model considers most important in a sentence for its decision, and second, Adversarial Attacks (such as HotFlip and Input Reduction), which introduce small changes to the text to test whether the model is easily misled or still produces a correct prediction. This tool aims to help researchers see where models tend to make mistakes. For example, sometimes models focus only on shared words between two sentences, even when their meanings differ. In some cases, the model cannot determine the correct meaning of words with multiple senses from the sentence context and keeps its initial incorrect interpretation. The model may also rely on shallow, irrelevant but frequent features (spurious patterns) instead of truly understanding the concept, which leads to incorrect predictions. By highlighting influential words, AllenNLP reveals these weaknesses and supports error analysis and the identification of potential biases. However, the outputs of AllenNLP are not always a reflection of the model’s “true understanding”; rather, they show which parts of the input the model is more sensitive to. Gradient-based methods can be noisy; they mostly capture local sensitivity and do not provide a full interpretation of a transformer model’s internal mechanisms. Moreover, in modern models such as BERT, where each word representation is formed contextually through interaction with other words, surface-level changes and simple lexical substitutions used in traditional adversarial attacks do not necessarily change the model’s decision and therefore tend to be less effective. Therefore, results obtained from this tool should be interpreted with caution and used alongside manual analysis, more detailed qualitative evaluation, and a broader range of test scenarios.

**Figure 17:**
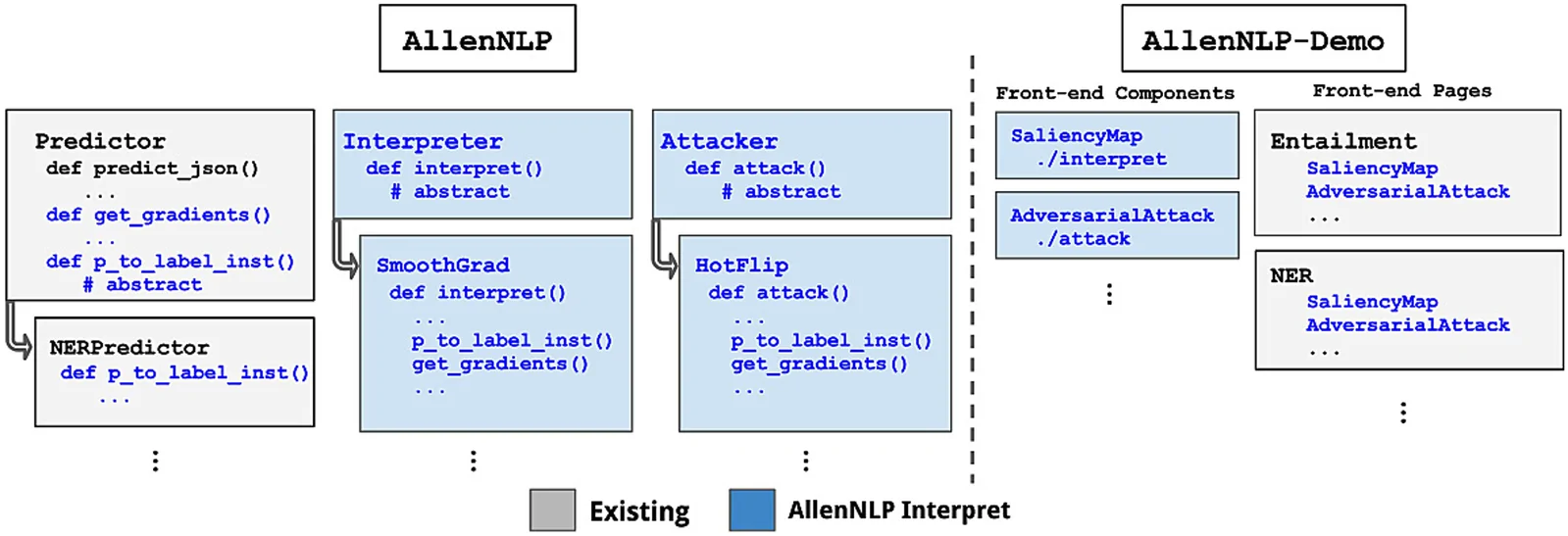
System overview of the AllenNLP interpretability toolkit [85]. This figure summarizes both the back-end components (Predictor, Interpreter, Attacker, SmoothGrad, and HotFlip; left) and the front-end modules (SaliencyMap and AdversarialAttack; right) integrated within the AllenNLP and AllenNLP-Demo ecosystems. Functionality introduced by the interpretability toolkit is distinguished from existing components, illustrating how gradient-based and adversarial methods are exposed to end users through reusable visualization pages.

As shown in Fig. 18, exBERT [86] is an interactive tool for visualizing and analyzing learned representations in transformer-based models, particularly BERT. This tool provides three main views—the attention view, the corpus view, and the summary view—which enable a comprehensive examination of model behavior. exBERT supports fine-grained token-level analysis through linguistic annotations (such as part-of-speech (POS) tags, named entities, and syntactic dependencies), interactive token masking, and nearest-neighbor search based on cosine similarity. These capabilities allow users to observe how attention patterns change when specific tokens are masked and to explore embeddings that are semantically similar within the corpus. Across different use cases, the tool demonstrates the evolution of BERT’s layer-wise representations. Lower layers primarily capture general features such as punctuation and basic syntactic dependencies, while higher layers encode richer semantic information and more complex syntactic relations (for example, verbs and their direct objects). In addition, exBERT enables attention-head analysis, identifying positional heads (those attending to neighboring tokens) and heads that encode specific syntactic relations such as possession. exBERT provides a deeper understanding of how transformer models learn, organize, and process linguistic information across different layers. However, exBERT is an exploratory tool that heavily relies on nearest-neighbor similarity based on embeddings and corpus examples; therefore, the insights provided are primarily qualitative rather than causal, and its interpretability is more influenced by the coverage, domain, and biases of the underlying corpus than by direct access to the internal mechanisms of the model.

**Figure 18:**
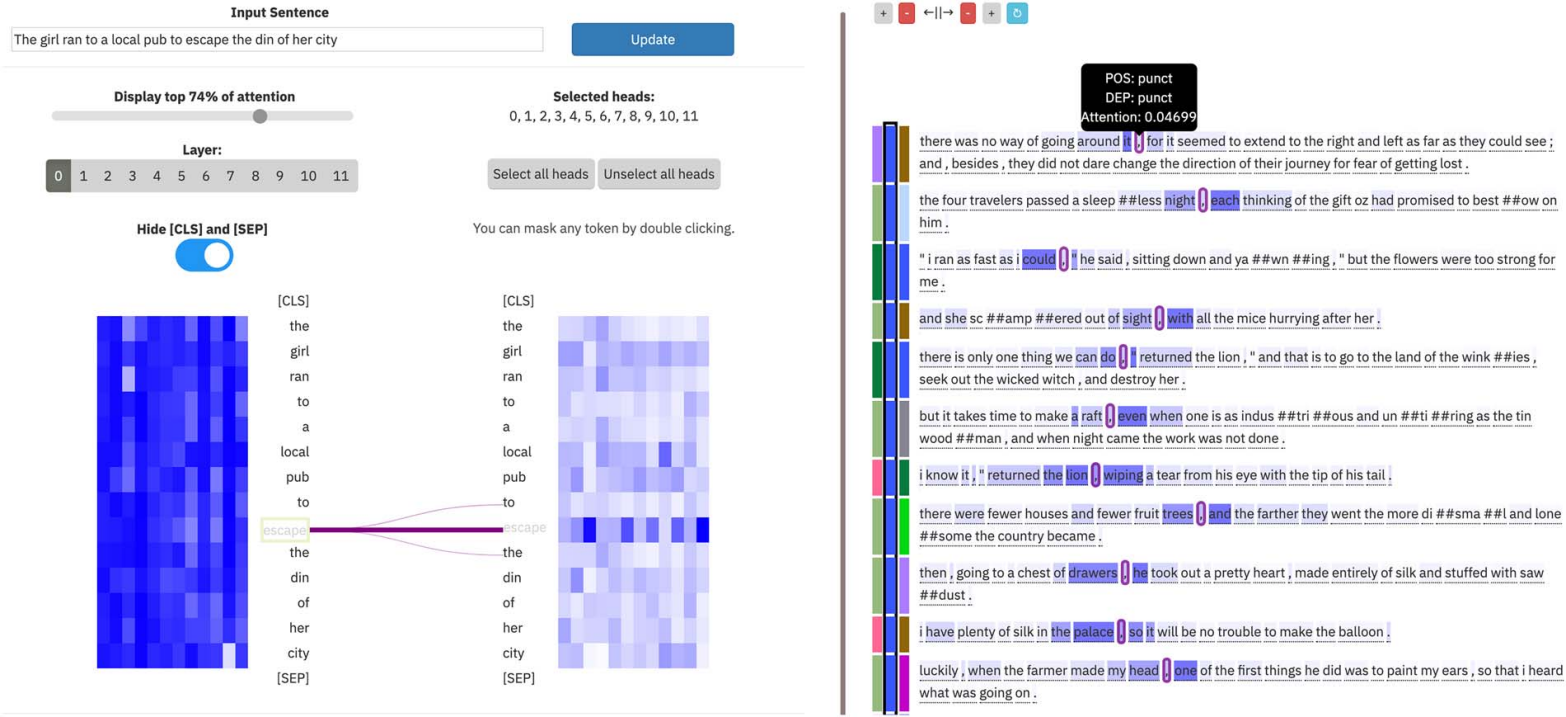
Overview of the exBERT interface [86]. The tool provides coordinated views for analyzing attention patterns and their occurrences across a large corpus. Users input a sentence in (a) to explore self-attention (c) and selectively mask tokens (b). A key feature is the corpus-level analysis (d–g), which allows researchers to search for similar attention patterns in annotated data, with linguistic metadata and summarized statistics (f, g) providing insights into model behavior beyond individual instances.

InterpreT [88] is an interactive visualization tool designed to analyze transformer models by tracking and displaying token representations across different layers (Fig. 19). In addition to aggregated layer-level views—including the head summary plot (b) and the average embedding distance plot (e)—the tool allows the exploration of token embeddings through t-SNE visualizations (d), making it possible to observe how syntactic and semantic relationships may emerge as representations evolve across layers. A summary table (a) provides token-level information such as layer indices and attention contribution, while attention matrices (c) visualize direct token-to-token interactions. InterpreT has been evaluated in two application scenarios: Aspect-Based Sentiment Analysis (ABSA) and the Winograd Schema Challenge (WSC). In the ABSA scenario, as shown in Fig. 19, InterpreT is used to examine differences in model performance across domains. The visualizations indicate that incorporating syntactic information in the LIBERT model—through a dedicated attention head that tracks syntactic relations between words—can help the model better capture sentence structure. This may reduce the effect of domain shift between different sentiment analysis datasets. In fact, the observed attention patterns suggest that syntactically aware heads may be associated with greater model robustness to domain changes. In the WSC application, InterpreT is used to examine pronoun resolution by visualizing attention patterns between coreferent and noncoreferent terms. By displaying token embeddings in reduced-dimension spaces such as t-SNE across different layers of the model, the tool enables comparison of how representations are distributed. Observations show that in deeper layers, the embeddings of coreferent tokens tend to move closer to each other in this space, while unrelated tokens become more distant. This pattern may indicate gradual changes and the formation of semantic and syntactic relations in representations across the layers of the model. Overall, InterpreT provides an intuitive way to observe and analyze linguistic patterns at different levels of transformer networks. Since InterpreT is a visualization-based tool, its analyses rely on observing embeddings and data examples. For this reason, its results are mostly descriptive and qualitative, and they do not directly reveal the internal decision-making mechanisms of the model. In addition, these analyses depend to some extent on the quality of the representations and the limitations of dimensionality-reduction methods.

**Figure 19:**
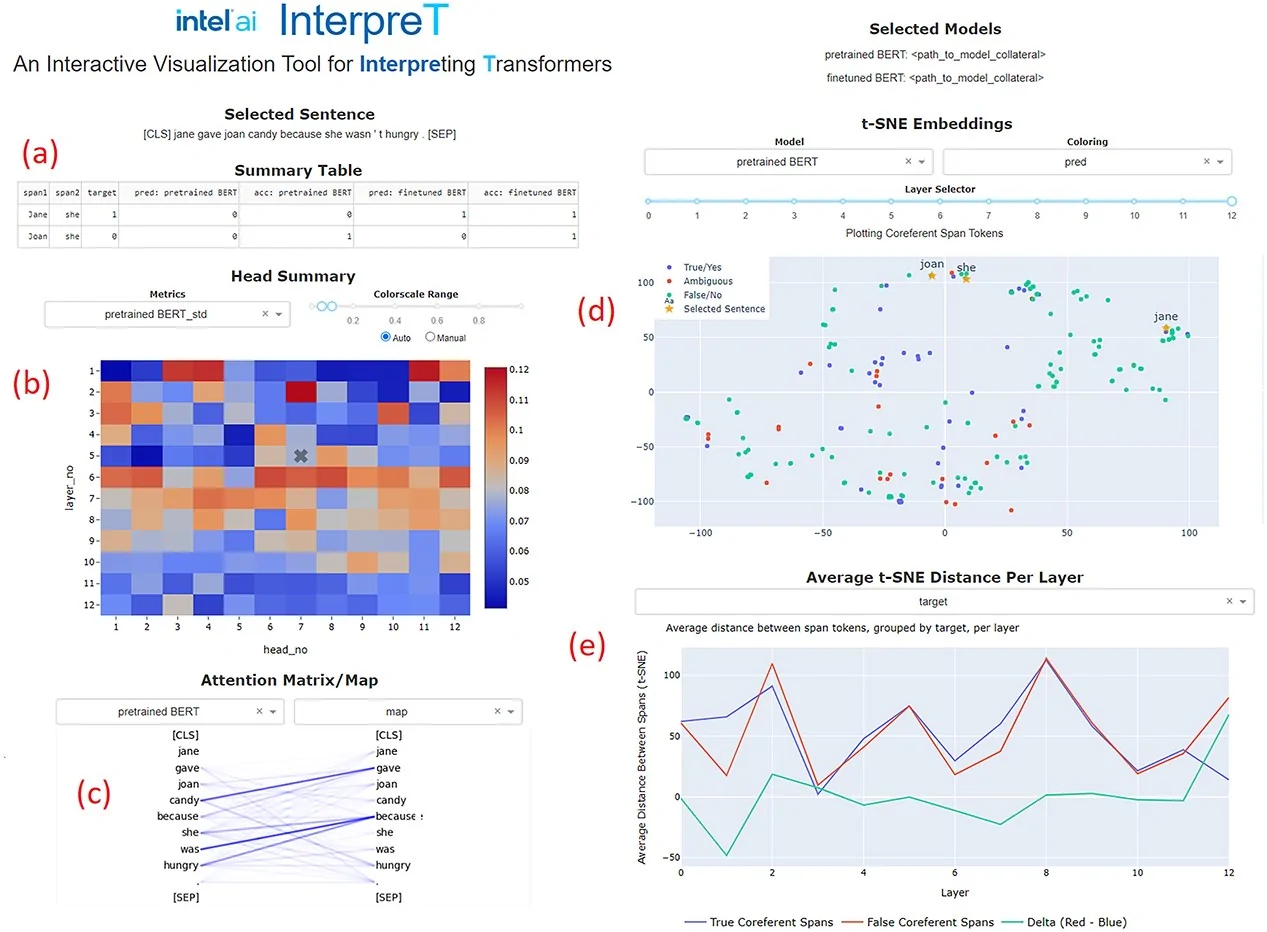
The InterpreT interactive visualization tool [88]. The interface provides coordinated views for analyzing transformer models. (a) Summary Table: Displays task-specific information for the selected input. (b) Head Summary Plot: Visualizes statistics of attention heads across layers. (c) Attention Matrix/Map: Shows token-level attention patterns. (d) t-SNE Embedding View: Illustrates contextualized token representations, revealing changes in spatial relationships**.** (e) Average t-SNE Distance Plot: Tracks the average distance between token embeddings across different layers. These panels collectively enable a deep dive into how transformers process and represent information.

T^3^-Vis is an interactive environment for better understanding Transformer models [89]. This tool helps us see how the model behaves at different levels—from the overall data representation to layers, heads, and even individual tokens. As shown in Fig. 20, T^3^-Vis consists of four coordinated views: a Projection View (A) for observing general representation patterns, a Data Table (B) for textual details and labels, an Attention Heads View (C) for comparing attention heads, and an Instance Investigation View (D) that allows for a deep dive into a specific input. These views are interconnected; by selecting or filtering an instance, the other views update simultaneously, providing the user with a complete analytical framework from general overviews to fine-grained details.

**Figure 20:**
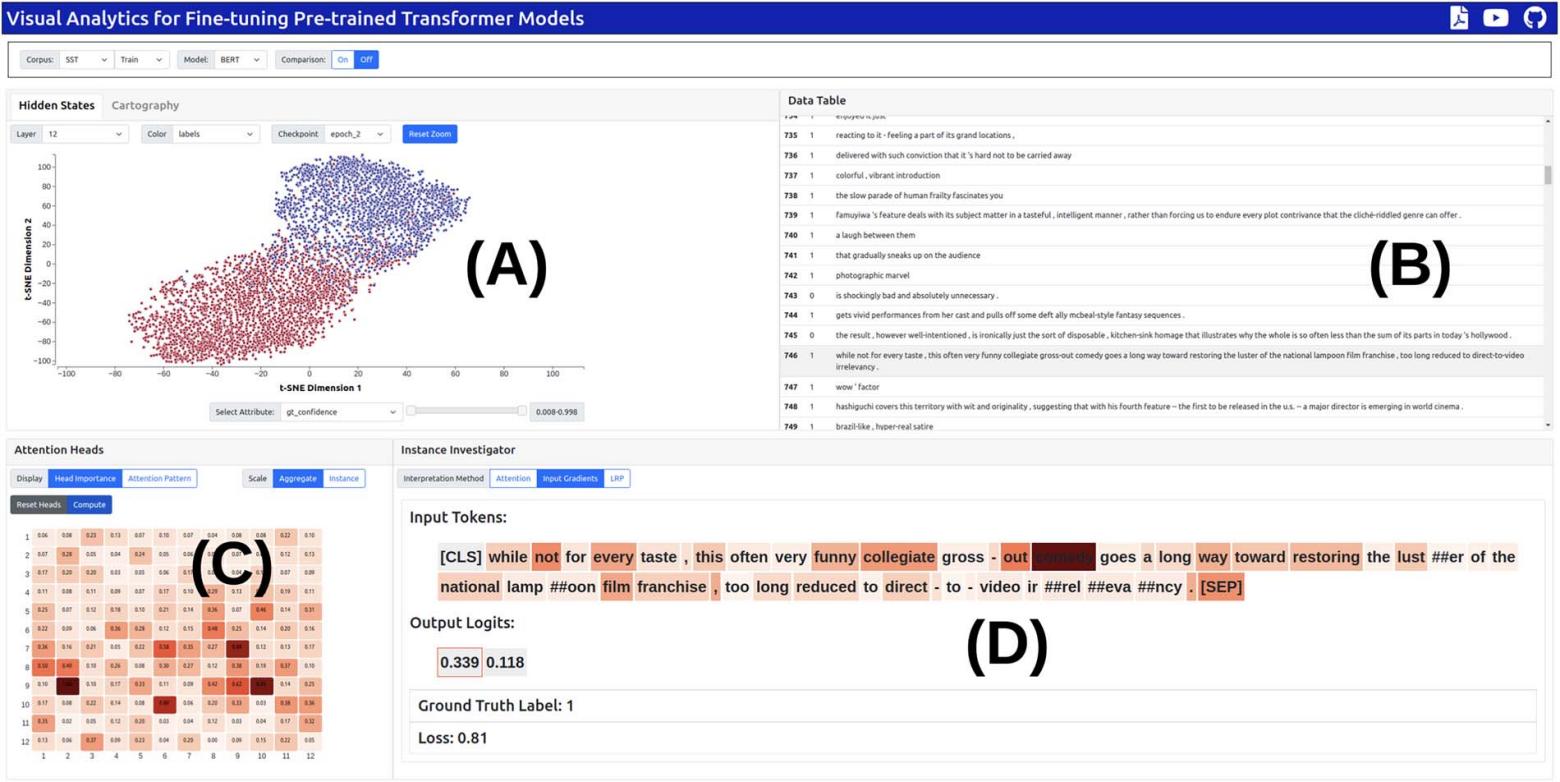
Overview of the T^3^-Vis interface [89]. (A) Projection View provides a global dataset-level overview; (B) Data Table lists instances and associated metadata; (C) Attention Head View enables selective head importance assessment and pruning; (D) Instance Investigation View facilitates detailed examination of model decisions on individual sequences, including attention weights and attribution methods.

The capabilities of T^3^-Vis are demonstrated through two main case studies in the paper. In the first case, “Pattern Exploration for an Extractive Summarizer,” we can observe how the model focuses on important text tokens across different layers and heads. In the second case, “Error Analysis for Topic Classification,” the tool is used to examine model errors. This allows the user to filter instances based on ground-truth labels and model predictions, isolate misclassified examples, and identify the sources of recurring errors, including those stemming from a lack of domain knowledge. A key advantage of T^3^-Vis over tools like BertViz or LIT is its ability to assess attention head importance and perform selective head pruning to evaluate how removing each head affects the model’s output.

However, it should be noted that T^3^-Vis is designed for fine-tuned models, as it relies on model predictions and ground-truth labels to analyze behavior. The paper does not provide a strictly theoretical definition for the “head importance” metric; rather, it is a computed metric that helps the analyst spot patterns, though it does not fully explore the role of each head in the model’s reasoning. Nevertheless, T^3^-Vis provides a good space for researchers to analyze the relationship between the model’s structure and its output within the context of a specific task.

Despite these advantages, T^3^-Vis does not cover all aspects of the model’s mechanics. It focuses on labeled instances and attention head analysis, and it does not address more fundamental questions such as evaluating pretrained models, causal analysis of decisions, or tracking the model’s behavioral evolution throughout the entire training process. These gaps show that while T^3^-Vis is efficient for interactive head-level analysis, it does not yet explain all dimensions of model behavior—which could serve as an interesting path for developing more precise tools in the future.

Dodrio [90] is an interactive visualization tool (Fig. 21) for interpreting attention mechanisms in transformer-based models. It helps researchers and practitioners analyze model performance through the lens of linguistic knowledge. Unlike many interpretation tools that focus only on attention weights, Dodrio integrates these weights with both syntactic and semantic information. As shown in Fig. 21, the tool uses an overview-plus-detail interaction model, which includes the Dependency View (A) for exploring syntactic relations, the Semantic Attention Graph (B) for visualizing attention distribution and node saliency, and the Attention Head Overview (C), which provides a global view of all heads across different layers. A key analytical feature of this tool is its encoding of linguistic properties: colors signify semantic or syntactic alignment, and circle size denotes the importance score of each head (D). Additionally, Dodrio enables comparative analysis, such as examining how linguistic knowledge evolves across layers or comparing different model architectures (like BERT versus DistilBERT). This provides deeper insights into model decisions, particularly when using linguistically annotated corpora.

**Figure 21:**
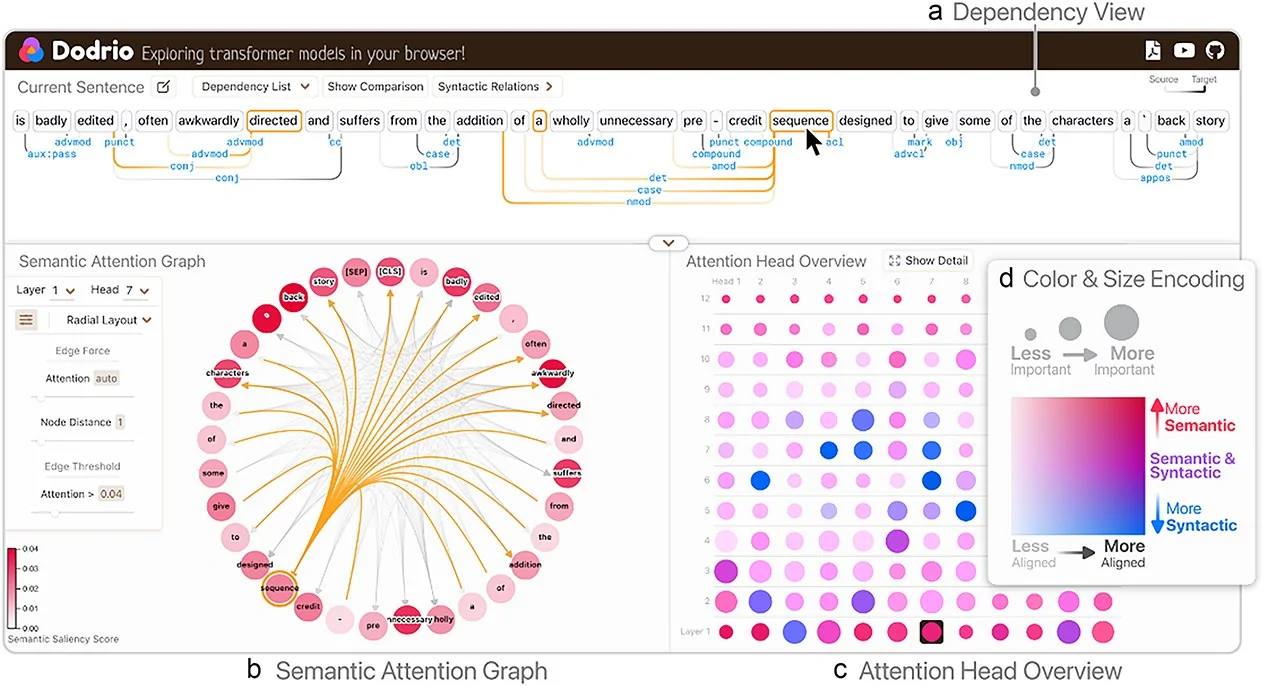
The Dodrio interface illustrating the exploration of attention weights from a fine-tuned BERT model on the SST2 dataset [90]. (a) The Dependency View displays syntactic relationships as directed arcs, with the arc direction indicating source and target tokens. (b) The Semantic Attention Graph visualizes attention distribution using a radial layout, where nodes represent tokens and line prominence indicates saliency. (c) The Attention Head Overview provides a grid-based global perspective of all heads across layers. (d) A visual encoding distinguishes semantic and syntactic alignment, while circle size denotes each head's importance score in the model.

Dodrio is a valuable and feature-rich tool, but it also has limitations that researchers could address to develop more comprehensive solutions. Its effectiveness heavily depends on the availability of high-quality linguistic annotations. Furthermore, although it does a great job of visualizing the connection between attention patterns and linguistic features, it remains primarily a visualization tool. Dodrio focuses on identifying correlations between model behavior and linguistic rules, but it does not provide a way to explain the causal processes behind the model’s decisions. These points suggest that while Dodrio is a powerful framework for interactive analysis, it works best when used alongside other interpretability methods.

The Provis tool analyzes protein-based Transformer models and visualizes their functional and structural features by examining attention mechanisms [91]. This study demonstrates that attention identifies high-level protein features and meaningful relationships between amino acids that are distant in linear sequence but proximal in the 3D structure. This is illustrated in Fig. 22a, where attention heads in a de novo designed TIM-barrel protein (PDB: 5BVL) focus on residues that are distant in the sequence yet physically close in space. Specifically, there is a strong correlation between attention patterns in certain heads and contact maps, indicating that attention weights are significantly aligned with amino acid contacts. Binding sites are another core focus of this research. The study shows that multiple attention heads tend to focus on these binding regions. A clear example of this at the residue level is shown in Fig. 22b, where attention is focused on G27 in the HIV-1 protease (PDB: 7HVP), a region critical for the binding of small-molecule inhibitors. This claim is examined across four models: ProtAlbert, ProtBERT, ProtBert-BFD, and ProtXLNet. Among these, ProtAlbert shows the strongest focus on binding sites, whereas ProtXLNet demonstrates less emphasis on them.

**Figure 22:**
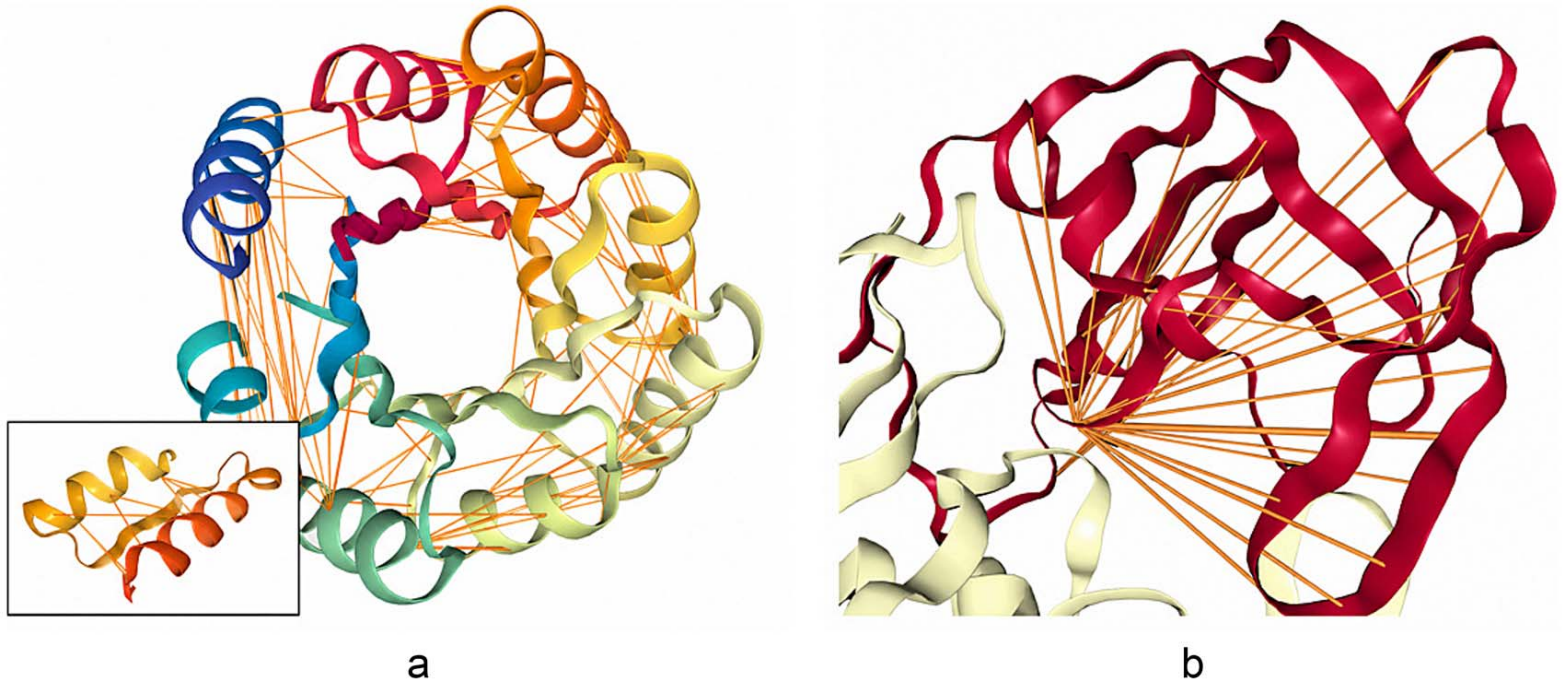
Visualization of biological interpretability in protein Transformers using Provis [91]. Lines indicate attention between amino acids, with line thickness proportional to attention weight (values below 0.1 are hidden). The figure demonstrates how specialized heads recover (a) high-level structural contacts and (b) functional binding sites without explicit supervision. (a) Structural Interpretation: Attention in layer 12, head 4 targets amino acid pairs that are spatially proximate in 3D space (see inset subsequence 117D–157I), despite being distantly located in the primary sequence. This is exemplified in a de novo designed TIM-barrel (PDB: 5BVL), where the model recovers the protein’s characteristic structural symmetry based solely on pretraining [91]. (b) Functional Interpretation: Attention in layer 7, head 1 targets binding sites, a key functional component. In HIV-1 protease (PDB: 7HVP), the attention is centered on residue G 27, which is a critical site for the binding of protease inhibitor small-molecule drugs [91].

These findings highlight the ability of the attention mechanism to encode functional regions of biological significance. Among the identified biological signals, structural contacts have a major impact on attention patterns. Evolutionary relationships found in amino acid substitution patterns also emerge indirectly through correlations with substitution matrices. This paper also explores cross-layer dynamics and mentions parameter sharing between layers in the ProtAlbert model. It utilizes diagnostic classifiers to relate attention weights to biological information. The relationship between each pair of amino acids and their Pearson correlation with BLOSUM shows that attention is consistent with substitution patterns. Provis suggests that these models do not merely store the identity of amino acids but also learn the biochemical relationships between them. In summary, Provis provides a structured framework for interpreting how Transformer models encode protein structure, binding behavior, and relationships between amino acids, both at the residue level and the residue-pair level.

Despite the capabilities of Provis, it is necessary to consider several points regarding the analyses presented in this paper. First, the core approach is based on “correlation” and “alignment,” which should not be interpreted as a proof of “causality.” Second, this study focuses primarily on pretrained models, and its efficacy on fine-tuned models requires further investigation. Third, when evaluating the capabilities of protein models via Provis, it should be noted that the success of probing classifiers in extracting biological information indicates that these models have learned these structural and functional features during self-supervised pretraining and have encoded them within their representations. Provis attempts to uncover the nature of some of these features by defining specific scenarios. However, it should be noted that the results of these evaluations reflect only the portion of the model’s knowledge that aligns with the chosen benchmarks (such as contact maps or binding sites). Therefore, the absence of a specific feature in Provis does not necessarily imply its absence in the model; it may instead stem from the insufficiency of the classifier used or a lack of coverage in the available benchmarks. Thus, Provis does not define the absolute boundaries of the model’s potential but rather serves as a tool for identifying the model’s capabilities. Finally, given the authors’ warnings regarding the potential for “hidden biases” and “spurious correlations” in Transformers, the use of Provis should be complemented by other analysis tools to prevent misinterpretation.

LIT is an open-source, interactive platform designed for the analysis, evaluation, and interpretation of NLP models [87]. As illustrated in Fig. 23, the LIT integrates various perspectives—such as embedding projections, data tables, and a datapoint editor—to facilitate interactive model debugging and counterfactual analysis. Developed by Google Research and validated with models like BERT and T5, LIT is a framework-agnostic tool that supports a wide range of classification, sequence-to-sequence (seq2seq), and structured prediction models. It allows users to investigate model behavior through multiple lenses, including predictions, attention mechanisms, saliency maps, and confusion matrices. A standout feature is the Datapoint Editor, which enables users to perform controlled perturbations to the input, thereby assessing the robustness of model decisions.

**Figure 23:**
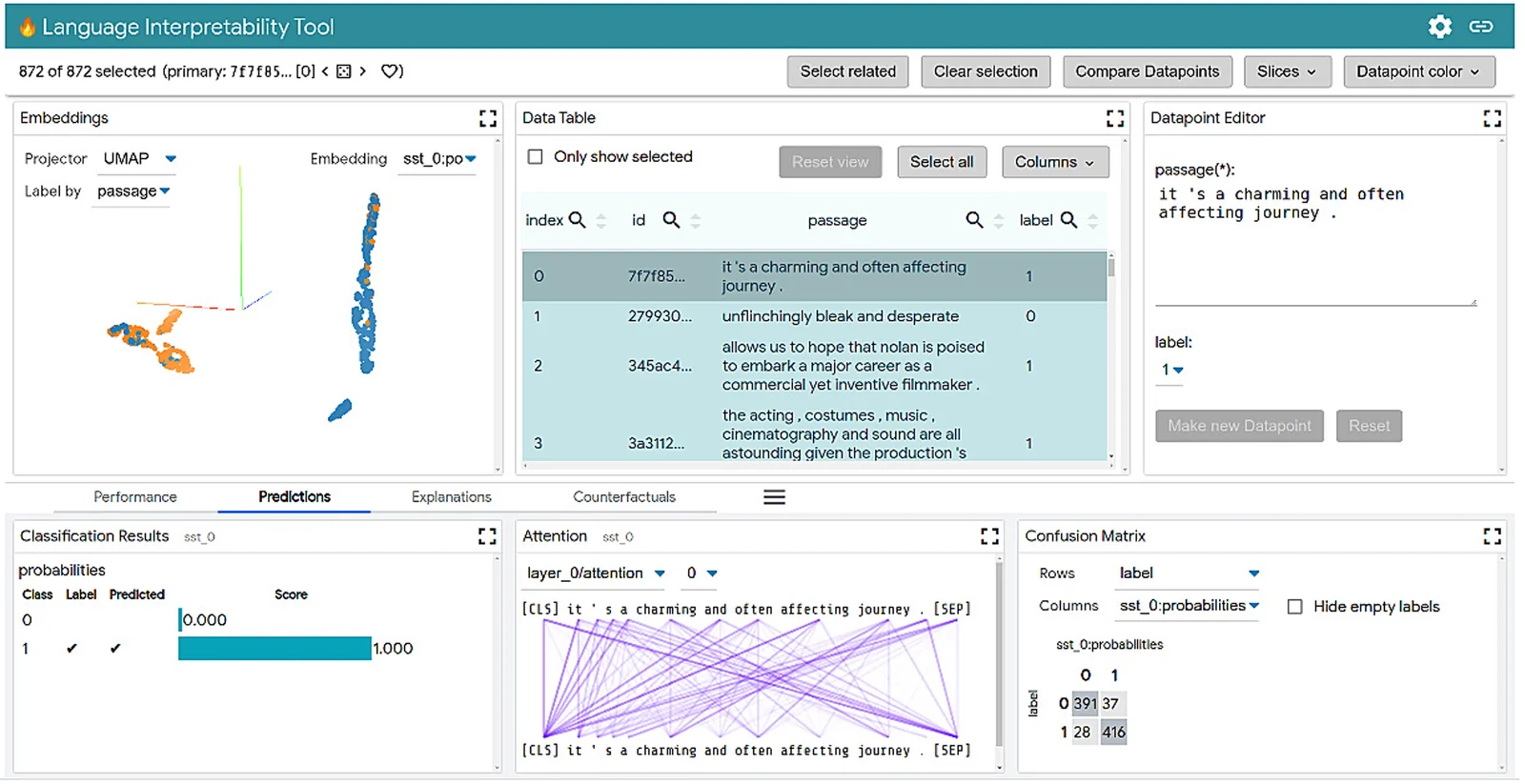
Overview of the Language Interpretability Tool (LIT) interface [87]. The user interface (UI) combines coordinated views for model inspection, including an embedding projector, a data table, and a datapoint editor for controlled input perturbations (top). The lower panels provide complementary analysis modules such as prediction summaries, attention visualization, and a confusion matrix, enabling interactive debugging and robustness assessment through counterfactual edits.

LIT has been widely applied to analyze complex linguistic scenarios, such as understanding the role of negation in sentiment analysis and identifying gender biases in coreference resolution. By enabling counterfactual analysis, the tool allows researchers to perturb inputs in a controlled manner to test model stability across different conditions. This approach helps determine whether a model has truly learned linguistic logic or is merely relying on repetitive, irrational patterns (spurious correlations) in the training data. Ultimately, LIT serves as a precise model validation instrument rather than a mere visualization tool. It acts as an efficient complement to methods that focus on internal representations.

Although LIT offers significant flexibility and a modular design, it faces certain limitations. First, it is primarily an evaluation tool and is not intended for real-time monitoring during training. Due to its framework-agnostic nature, it lacks the deep, tight integration with specific architectures that is typically found in framework-native tools like AllenNLP Interpret. Furthermore, as an interactive, browser-based tool, it is constrained by memory overhead, with performance typically limited to ~10 000 data points per session. Consequently, while LIT is highly effective for rapid exploration and hypothesis testing, it may prove insufficient for comprehensive model analysis without the use of sampling or data aggregation techniques.

From a biological perspective, tools like Provis help researchers analyze the internal representations of protein language models [91]. These tools highlight “attention patterns” that correspond to actual structural contacts or binding sites in proteins. This allows researchers to interpret the model’s decisions in a biological context, which can help in discovering new patterns in biological data.

In this regard, diagnostic tools such as LIT [87] and InterpreT [88] are also very useful. By using methods like “perturbation” and “attribution,” these tools help identify false correlations within the model. Additionally, tools like BertViz [84], AllenNLP Interpret [85], and exBERT [86] provide further insights for examining the model’s layers in detail. On the other hand, specialized frameworks like Provis [91] focus on ensuring that the model’s internal representations are based on valid biological principles. Together, these tools allow researchers to verify that the model is relying on genuine biological signals, which increases confidence in its predictions.

Despite these advances, extracting biological features from transformer models remains a challenge. Structural and evolutionary features are often learned implicitly during “pretraining,” making them easier to access. However, extracting functional signals is much harder because these signals are global and arise from complex dependencies that are not easily visible in standard attention mechanisms.

This technical challenge leads to what we call the Significance Gap. This is a disconnect between the model’s numerical outputs (like attention weights) and the biological explanations scientists need for their experiments. For example, a model might correctly predict a protein’s drug resistance but fail to explain why—such as identifying exactly which change in a binding site caused the resistance. Since these details are critical for laboratory validation, current interpretability tools need to evolve to bridge the gap between numerical predictions and biological mechanisms. In this context, the TGDS framework, proposed by Karpatne *et al.* [12], offers a clear roadmap. This paradigm suggests that for reliable scientific discovery, models should not only rely on raw data but also be guided by prior biological knowledge, ensuring their outputs are biologically meaningful and trustworthy.

The tools reviewed demonstrate the multi-dimensional nature of transformer interpretability, ranging from attention pattern observation to complex counterfactual analyses. Given that most models and tools developed for NLP are extensible to biological data, this section explores whether interpretability tools can effectively transition to the biological domain. However, transitioning these tools to the biological domain reveals a significant gap between general linguistic patterns and specialized biological mechanisms. As summarized in Table 5, the practical utility of an interpretability framework for bioinformatics depends heavily on its capacity to handle nonlinear structural dependencies, high-dimensional protein embeddings, and task-specific biological priors.

**Table 5:** Comparative analysis of transformer interpretability tools.

Tool	Focus area	Bio-adaptability	Practical constraints	Key limitation
BertViz	Attention patterns	Moderate (local)	Visual complexity	Diagnostic signal only
AllenNLP	Sensitivity/adversarial	Low (text centric)	Linguistic annotation	Gradient noise
exBERT	Learned representations	Moderate (exploratory)	Training data dependency	Qualitative/noncausal
InterpreT	Layer-wise embedding	Moderate (structural)	Dimensionality reduction	Descriptive/nonanalytical
T^3^-Vis	Error analysis/coordinated views	High (customizable)	Fine-tuned model need	Heuristic importance metric
Dodrio	Attention/linguistic knowledge	Moderate (syntactic)	Annotation intensive	Correlation focused
Provis	Functional/structural features	Very high (domain specific)	Pretrained model need	Spurious correlations
LIT	Counterfactual analysis	High (flexible)	Browser memory limits	Scalability/evaluation centric

In Table 5, we provide a comparative synthesis of transformer interpretability tools, focusing on their applicability to biological research. The content of the “Practical Constraints” and “Key Limitation” columns has been extracted and collected from the original publications and documentation of these tools (with appropriate citations). The “Bio-Adaptability” column reflects our inference regarding how well each method aligns with common bioinformatics requirements. Based on this comparative framework, we further examine the key challenges and trade-offs in selecting interpretability tools for biological applications. While tools such as BertViz and AllenNLP provide essential diagnostic signals for general tasks, the inherent complexity of biological data poses significant challenges to these tools. In contrast, frameworks such as Provis and T3-Vis offer greater domain adaptability, yet they often require specialized setups or fine-tuned models. Therefore, choosing an interpretability tool for biological applications involves a strategic trade-off between the level of structural integration and operational overhead. Future developments in this field must transition from correlation-based observation to causal inference, explicitly incorporating the hierarchical and nonlinear properties of biological data into the interpretability process to bridge existing gaps.

#### Functional categorization and multi-dimensional comparison of explainability tools for transformer-based models

In the previous section, explainable tools for transformer-based models were introduced. In this section, we provide a structured comparison between the tools from different aspects. In the classified table, potential use cases of the tools, visualization strategies, and interpretability details are discussed. To provide a more straightforward overview of the capabilities of explainability tools for transformer-based models, Table 6 categorizes the tools by use case. This categorization is based on a thorough literature review of each tool. In some cases, rather than being explicitly mentioned in the primary sources, the results are the outcome of inferential analysis and examination of the tools’ performance in terms of their potential functions. Therefore, the present table not only covers the tools’ obvious use cases but also highlights their implicit and adaptable tasks across different domains.

**Table 6:** Use case classification based on observed and potential capabilities of explainability tools (author-defined taxonomy, entries compiled from primary sources)

Description	Related tools	Use case
Identifying model prediction errors and finding their causes	AllenNLP, T-Vis, DODRIO, LIT	Model debugging
Exploring attention relationships, heads, and semantic correlations	BertViz, ExBERT, Provis, InterpreT	Understanding attention patterns
Detecting different biases	BertViz, AllenNLP, InterpreT. LIT	Bias analysis
Analyzing hidden representations of tokens/sequences	InterpreT, T-Vis, Provis	Embedding interpretation
Comparing different models or versions on the same task	exBERT, DODRIO	Comparative model analysis
Evaluating model performance between different domains or contexts	InterpreT	Cross-domain evaluation
Structure prediction analysis, function, or amino acid similarity in biological data	Provis	Bio-specific use, understanding attention patterns, embedding interpretation
Identifying influential input features and decision logic	AllenNLP	Decision rule analysis
Analyzing and explaining errors by tracing influential training examples	LIT	Generation error analysis

We review the information in Table 6 here. The tools, such as LIT and InterpreT, are organized into several categories. It indicates their flexibility in support of a variety of interpretability tasks. In contrast, tools such as Provis are only available for specific bioinformatics applications, with capabilities including structure prediction and embedding analysis. This table can guide researchers in selecting tools suited to their particular analytical goals, such as debugging, bias detection, interpreting hidden states, or comparing models. To explore the tools in more depth and facilitate a clearer understanding of how the internal mechanisms of transformer-based models are represented and interpreted by different explainability tools, Table 7 categorizes the tools by view type. Each view type represents a distinct visualization strategy (for example, attention matrices, embedded images, and semantic graph representations). While some tools may support multiple views, they are grouped by the view type that best characterizes their main functionality. In addition to highlighting the primary visualization capabilities of each tool, this table also clarifies the ability of these views to interpret the model at different levels—token, layer, corpus, or semantic structure.

**Table 7:** Categorization of transformer explainability tools by visualization view type (author-defined taxonomy, entries compiled from primary sources)

Description	Tools	View type
Shows token attention between heads and layers	BertViz, ExBERT, AllenNLP, InterpreT, T3-Vis, DODRIO, Provis, LIT (Attention Visualization)	Attention View
Analyzes neuron activations and their correlations with linguistic or semantic features	BertViz	Neuron View
Enables large-scale exploration of model behavior across multiple examples or datasets	exBERT, LIT (Data Table)	Corpus View
Projects token/sentence embeddings into 2D space for analyzing semantic similarities	InterpreT, T3-Vis, LIT	T-SNE/Embedding View
Displays semantic structures among tokens using attention-based graph visualization	DODRIO	Semantic Graph View
Presents layer-wise visualization of model behavior including attention, similarity, and importance matrices	Provis	Matrix View (Layer-wise)
Highlights which parts of the input most influenced the model’s decision	AllenNLP, LIT	Saliency View
Allows editing inputs to observe how model predictions change under small perturbations	LIT	Counterfactual View
Analyzes model performance using metrics, confusion matrix, and error breakdowns	LIT (Confusion Matrix, Metrics Table, Error Analysis, Model Comparison, Classifier Results)	Performance/Error View

A comparative overview of several explainability tools for transformer-based models is presented in Table 8 (summarizing each tool’s application domain, the models on which it has been tested, and the level of explanation it provides). The “Level of Explanation” column indicates whether the tool offers interpretability at the token, layer, head, or instance level, or employs a multi-level approach (e.g. token-sentence-summary or token-pair analysis). Additionally, this table indicates whether the tools have been applied to general NLP or domain-specific areas such as Bio. This classification enables researchers to select the most relevant and suitable tool for their model’s specific properties.

**Table 8:** Comparison of transformer explainability tools by application domain, analyzed models, and explanation granularity (author-defined taxonomy, entries compiled from primary sources.)

Tools	Application domain	Analyzed models	Level of explanation
[LIT]	NLP	Bert, T5, Other NLP models (classification, seq2seq, structured prediction)	Token, layer, head, span, sentence, instance
Bertviz	NLP	Bert, OpenAI GPT-2	Token, head, neuron
AllenNLP	NLP	Bert, RoBERTa	Token
exBERT	NLP	Bert	Token, layer
InterpreT	NLP	Bert, LIBERT	Token, layer
T-Vis	NLP	BertSum, RoBERTaSum, Bert	Token, sentence, summary
Dodrio	NLP	Bert, DistileBert	Instance
Provis	Bio	Bert, ALBERT, XLNet	Token-pair, Head, Layer, Cross-layer, Embedding

Table 8 can help us choose a tool. Tools such as LIT and Provis offer multi-level interpretability that can cover a wide range of linguistic and structural units (e.g. from tokens to instances or embeddings). For a comprehensive understanding of models and interpretation of complex tasks, LIT and Provis are good choices. In contrast, tools such as AllenNLP and exBERT provide only token-level or layer-level insights, which may limit their use in biological sequence analysis, where cross-layer or relational representations (e.g. token pairs) are critical. Notably, Provis is the only bioinformatics tool to support cross-layer and embedding-level interpretations—a functionality largely absent in NLP-focused tools. It represents a gap in domain-specific interpretability tools that go beyond NLP.

Building on the comparative analysis presented in Tables 5–8, it becomes evident that despite significant progress, current transformer interpretability tools face serious challenges. The first gap is the difference in interactivity. Unlike advanced NLP tools like LIT, which allow for real-time exploration of model decisions through dynamic and counterfactual analysis, specialized bioinformatics tools like Provis mostly focus on static visualizations. These static images prevent biologists from gaining a deep and causal understanding of how the model reasons. Furthermore, because biological data are more complex than text, these tools often face heavy computational costs, which makes it impossible to provide real-time responses. Also, these tools are often specialized for specific domains. For example, changing the input—from protein 3D structures to RNA sequences or other genetic data—can strongly affect their performance and accuracy. Consequently, these tools should not be used as sources for definitive proof, but as assistants for researchers to generate hypotheses in an interactive environment. For the future, bioinformatics needs a new generation of tools that combine two key features: first, interactive capabilities (for the user to test the model’s behavior in real-time), and second, a deep focus on biological structures (to understand the biological meaning of the model’s decisions). Developing tools that can perform these interactions at high speed and for large amounts of data (scalability) is a necessary step for the future of this field.

#### Limitations and future directions of explainability tools in transformer-based models

Despite progress in interpretability tools for transformer models, a review of existing tools reveals significant gaps in addressing user needs, particularly in computational biology and drug design. The primary focus of the tools is on representing attention weights, and they often display the attention structure as 2D maps. However, the features extracted at each layer are shown visually, without providing explicit information about their type (local or global, syntactic or semantic). It is not clear in which layers the model relies more on local information and in which parts global or semantic features are extracted. Moreover, many tools provide only a superficial view of the model’s behavior and lack more detailed analyses that can trace the process by which the model changes the nature of the features from syntax-based to semantics-based. There is still no tool that can map the features extracted in each layer to linguistic or cognitive dimensions in a comprehensive and explainable way. Another point is that existing tools primarily focus on specific models, such as BERT or RoBERTa, and do not provide a comprehensive view of the behavior of other models (e.g. lighter or domain-oriented models, such as biological models). Although some tools can load arbitrary weights, in practice, the graphical interface and interpretation logic are often dependent on the structure of a particular model. It reduces the generalizability of the tools to new architectures or custom models. Many of these tools cannot analyze the model internally and fall into the *post hoc* category; that is, they perform their analysis after the model has been trained without affecting the learning process. Although this approach is helpful for rapid analysis, it cannot recognize or guide the flow of feature learning across different levels of the model. Finally, one of the main challenges in current tools is the lack of a unified approach to combining different visual views and use cases. Most tools offer one or more simple views, such as an attention-head view or a neuron view, and enable specific-case analyses; however, they are still a long way from combining views and moving beyond the display level to multilayer analysis. For this reason, the user must examine the views separately and manually interpret their relationships to gain a deep understanding of the model’s behavior.

The biggest weakness of current tools is the lack of real interaction with the expert human user. None of these tools can express their outputs in a scientific language understandable to a domain-oriented researcher (e.g. a biologist or bioinformatician). A biological researcher expects a model to tell him or her that the most essential part of the protein in this prediction is a specific region in the chain that corresponds to a binding site. Alternatively, in more advanced applications, such as drug design, this part of the protein plays a minor role in the model’s decision-making. It could be a good candidate for mutation to study its effect on protein function. Such a level of explanation requires a connection between the model’s processing levels and biological concepts. This connection has not yet been structurally established in any existing tool. In the absence of such interactions, the tools remain more “demonstrative” than “explainable” and fail to serve as cognitive partners in the scientific process.

Consequently, future tools will need a more holistic approach to increase true explainability in a way that they can:

- Clarify the transition from one info level to another info level of language (syntax → semantics or sequence info → structure info),
- Analyze local/global features between layers,
- Support usability with diverse architectures without the need for a significant redesign,
- And provide multi-view and dynamic analytics in an interactive and understandable schema for nonspecialist users.

In recent years, SAEs (Sparse AutoEncoders) have garnered increasing attention as a promising approach for enhancing the interpretability of internal representations in transformer models [92]. The objective of these methods is to decompose dense neural activations into a set of latent features that are more sparse and, consequently, easier to interpret. Specifically, this approach has been proposed as a potential solution to the issue of feature superposition—a phenomenon where multiple concepts may be encoded simultaneously, either in the activity of a single neuron or in a shared activation pattern across several neurons [93]. By learning a dictionary of sparse features, SAEs can help reveal interpretable patterns within the model’s representations, some of which may correspond to meaningful linguistic, semantic, or biological structures in large transformer models [94]. Although this research area is still relatively nascent, preliminary results suggest that it may provide a promising path toward uncovering more structured and interpretable representations in large-scale models [92].

Another emerging challenge relates to the interpretability of MoE (Mixture-of-Experts) architectures, which have been increasingly employed in modern large language models to improve computational efficiency and scalability [95, 96]. In these architectures, a routing mechanism dynamically determines which expert network processes each token, thereby enabling a degree of functional specialization among experts [95, 96]. However, this dynamic routing raises new questions regarding interpretability, such as how routing decisions are made, whether experts truly achieve meaningful functional specialization, and how the allocation of tokens to experts influences model predictions. Since most existing interpretability methods were designed for dense transformers, they typically cannot fully account for the routing dynamics in MoE models. Therefore, the development of novel techniques for analyzing routing behavior and expert specialization in MoE models represents a key necessity and a primary direction for future research in interpretability.

Therefore, the future explainable tools should focus not only on the graphical and technical levels but also on the transition from mere representation to language-driven interpretation and on enabling active interaction with the human user. An interpretation that can be usable, reliable, and actionable not only for the machine learning engineer but also for the biologist, pharmacist, or physician. Only then can we transition from the representation stage to the stage of fundamental understanding. In this section, we examined the limitations of interpretability tools. In the next section, we will analyze the limitations of research in the two fields of NLP and bioinformatics and identify the existing gaps.

## Results and discussion

According to the studies reviewed in this survey, several important gaps remain in the interpretability of transformer-based models in NLP and bioinformatics, which may guide future research directions. In this section, we first discuss gaps related to NLP interpretability and then highlight open challenges in bioinformatics models.

### Gaps related to the interpretability of NLP models

One important issue is the analysis of bias in low-resource languages (that is, languages other than English). Previous studies have shown that most interpretability analyses have mainly been conducted on English datasets [97], and the behavior of models in low-resource settings has not yet been sufficiently examined [98, 99]. In particular, there is still a lack of deep interpretability methods for understanding how bias is formed under resource-limited conditions. Another important challenge is the discovery of deeper semantic relationships. Existing interpretability methods have mostly focused on relatively simple linguistic patterns, while more complex semantic phenomena such as metaphor, sarcasm, and humor have received less attention [100–103]. Although transformer-based models achieve strong performance on these tasks, the internal mechanisms behind this kind of semantic understanding remain unclear [104]. In machine translation models [105], interpretability studies have mostly focused on attention visualization and token alignment. However, only a limited number of works have examined how transformer models determine word order or maintain semantic consistency during translation. Understanding these mechanisms can provide deeper insights into multilingual semantic representation. Another challenge concerns the sensitivity of model outputs to small changes in the input [106]. Although previous studies have investigated the existence of this sensitivity, the internal mechanisms that cause these behavioral changes are still not understood in a comprehensive way. This issue is particularly important for improving the robustness and reliability of NLP systems. In question-answering (QA) models [107, 108], interpretability studies have traditionally focused on identifying which parts of the input text are attended to during answer generation [109]. However, such methods are often unable to reveal the underlying mechanisms of decision-making and reasoning [109]. Recent advances in mechanistic interpretability, such as the extraction of causal circuits, have begun to address this scientific gap by revealing how internal components (such as attention heads and MLP layers) interact to process content and perform reasoning [110]. These approaches show that going beyond simple input attribution is essential for a comprehensive understanding of how models prioritize information and reason over complex documents. The interpretation of multilingual transformer models is another important research gap. Existing studies show that multilingual models share information across different languages [98, 111], but the mechanisms that govern cross-lingual knowledge transfer and representation learning are still not fully understood from an interpretability perspective. Finally, the behavior of transformer models under the influence of noisy or erroneous inputs has still been examined less. For example, limited attention has been given to understanding how spelling mistakes or grammatical errors affect internal representations and prediction behavior [112]. Developing robust interpretability methods for noisy data can greatly improve the reliability of real-world NLP applications.

### Gaps related to the interpretability of bioinformatics models

Transformer architectures have recently achieved remarkable success in protein property prediction, revolutionizing computational biology [113]. Despite this progress, there remains a critical gap in our understanding of how these models effectively “unravel” the complex information encoded within amino acid sequences; specifically, limited studies have investigated how distinct biological properties are mapped to internal representations or specific attention heads [113]. Understanding these relationships could improve the biological interpretability of such models. Another important gap involves sequence generation for therapeutic and drug discovery purposes [114, 115]. Although transformer models can generate biologically meaningful sequences, the mechanisms underlying these predictions remain insufficiently understood. Improving interpretability in this area could increase the reliability of AI-assisted drug development. Bias in biological datasets is another critical issue. Previous studies have shown that biological data may contain different forms of sampling or population bias [116], which can directly affect model predictions. Addressing these biases is imperative, as failure to do so may lead to the propagation of net bias in biological models, ultimately undermining the reproducibility and credibility of AI-driven discoveries [116].

Nevertheless, limited work has focused on interpreting how these biases influence transformer-based biological models. Interpreting multi-omic models also remains a major challenge. These models integrate multiple biological data sources, such as genomic, transcriptomic, and proteomic information [117–119]. Beyond architectural complexities, there exists a profound gap in the interpretable integration of multi-omics data, where models must move beyond predictive accuracy to elucidate the biological rationale behind their decisions within the context of precision medicine [118]. Recent approaches, such as Pathformer, have begun to address this by incorporating prior biological knowledge into transformer architectures to enhance interpretability [119]; however, extending such pathway-informed mechanisms to a broader spectrum of multi-omics data and diverse temporal biological processes remains a critical research frontier. Although such architectures improve predictive performance, their internal decision-making processes are often difficult to interpret due to the complexity of multi-modal biological interactions. Less attention has been paid to the interpretability of time-series biological data. For example, transformer-based models designed for gene expression dynamics and temporal biological processes [120] require more robust interpretability techniques capable of explaining temporal dependencies and sequential biological behavior. Another challenge concerns transformer models that use multiple sequence alignment as input [121]. Although these models have significantly improved biological sequence analysis [122], interpreting how alignment information contributes to prediction decisions remains largely unexplored. Overall, despite the considerable progress of transformer models in bioinformatics, the interpretability of these systems is still limited by the inherent complexity of biological data and model architectures.

## Conclusion

This paper reviewed the methods and tools for explaining transformer models in two essential fields: NLP and bioinformatics. As transformer-based architectures become increasingly complex, explainability has become critical in research. The methods reviewed can be categorized into two types: norm-based and weight-based methods. Weight-based methods interpret the model from different perspectives according to the weights of its network nodes. Weight-based interpretation was further divided into gradient-based analysis, attention-based analysis, self-explanatory methods, probing-based analysis, and LIME-based analysis. Each method makes a significant contribution to model explanation and offers distinct advantages. However, they also face particular challenges. Most models focus on local parts of the model and pay less attention to the interaction between layers and their mutual influence. The tools presented often fail to generalize to new models and do not provide in-depth interpretations of the models. None of the existing methods provides a multi-level explanation that captures internal interactions. This challenge is particularly evident in multilayer transformer models, where attention is applied across multiple layers. Our analysis also identified several open challenges and research gaps—particularly in the interpretability of models used for low-resource languages, multi-omics data, time series, and biased biological datasets (e.g. protein datasets where the model learns to focus predominantly on well-annotated binding sites while underestimating other functionally important regions). These challenges underscore the need to develop more comprehensive, multi-level interpretability techniques that decrease the gap between deep models and interpretability methods. We aim to focus on these issues to support further advances in interpretable, domain-compatible transformer-based models for NLP and bioinformatics applications.

**Key Points**

- This review provides a comprehensive analysis of explainability methods in transformer-based models and focuses on their applications in natural language processing and bioinformatics.
- This paper categorizes and compares explainability techniques, identifies key tools, and assesses their strengths and limitations for explaining transformer models.
- This paper highlights the relationship between the increasing complexity of transformer models and the need for appropriate explainability methods to bridge the gap between model performance and human understanding.
- This paper explores key challenges in interpreting transformer models, including the difficulty of applying traditional explainability methods to highly complex models, and suggests directions for future research.
- This study emphasizes the importance of model explainability for building confidence in model predictions and ensuring clinical relevance, especially in healthcare and bioinformatics.

## Supplementary Material

Supplementary_materials_bbag447

## Data Availability

This study is a review article and did not generate any new data.
