# Supplementary material for: Explainability in transformer-based models: methods, tools, and applications in natural language processing and bioinformatics
Source: Brief Bioinform. 2026 Nov 12;27(6):bbag447. doi: 10.1093/bib/bbag447 (PMC13755357; doi:10.1093/bib/bbag447)
Supplement: Supplementary file 1 [file supplementary_materials_bbag447.pdf]

Statement of Significance

To help readers quickly grasp the core impact of this work, the following table provides a structured summary of its significance.

Element	Description
**1. Problem or Issue**	One of the most popular and widely used models across various fields, particularly in biomedical and bioinformatics research, is the transformer model. One of the most important concerns for researchers is trust in artificial intelligence models. This trust is achieved by interpreting transformer models. However, their complexity has created a gap between transformer models and their explainability and practical use in biomedical domains.
**2. What is Known**	Although interpretability methods have been widely studied in general NLP, recent reviews do not provide a structured and comparative overview that bridges both general NLP and biomedical/bioinformatics applications in transformer models.
**3. What This Paper Adds**	This article provides a comprehensive and structured review of interpretability methods for transformer-based models in NLP and Bioinformatics. Crucially, it introduces a novel adaptability analysis (as presented in Table 5) that examines the potential of established NLP tools for biological sequence analysis. The research categorizes various approaches, compares tools based on multiple criteria, and identifies critical research gaps in both Bioinformatics and NLP domains that require further investigation.
**4. Who Would Benefit**	Biomedical and bioinformatics researchers adopting transformer-based models will benefit from this structured review. It specifically assists practitioners in selecting the right interpretability tools for biological data, ensuring clinical relevance and model reliability. Furthermore, NLP researchers and AI developers will find this synthesis valuable, as it provides a rigorous comparative analysis of interpretability methods and tools that are fundamental to building transparent and trustworthy transformer models in any domain.
